# Gene expression in African Americans, Puerto Ricans and Mexican Americans reveals ancestry-specific patterns of genetic architecture

**DOI:** 10.1038/s41588-023-01377-z

**Published:** 2023-05-25

**Authors:** Linda Kachuri, Angel C. Y. Mak, Donglei Hu, Celeste Eng, Scott Huntsman, Jennifer R. Elhawary, Namrata Gupta, Stacey Gabriel, Shujie Xiao, Kevin L. Keys, Akinyemi Oni-Orisan, José R. Rodríguez-Santana, Michael A. LeNoir, Luisa N. Borrell, Noah A. Zaitlen, L. Keoki Williams, Christopher R. Gignoux, Esteban González Burchard, Elad Ziv

**Affiliations:** 1 Department of Epidemiology and Biostatistics, University of California, San Francisco, San Francisco, CA USA; 2 Department of Epidemiology and Population Health, Stanford University, Stanford, CA USA; 3 Department of Medicine, University of California, San Francisco, San Francisco, CA USA; 4 Broad Institute of MIT and Harvard, Cambridge, MA USA; 5 Center for Individualized and Genomic Medicine Research, Henry Ford Health System, Detroit, MI USA; 6 Berkeley Institute for Data Science, University of California, Berkeley, Berkeley, CA USA; 7 Department of Clinical Pharmacy, University of California, San Francisco, San Francisco, CA USA; 8 Department of Bioengineering and Therapeutic Sciences, University of California, San Francisco, San Francisco, CA USA; 9 Institute for Human Genetics, University of California, San Francisco, San Francisco, CA USA; 10 Centro de Neumología Pediátrica, San Juan, Puerto Rico; 11 Bay Area Pediatrics, Oakland, CA USA; 12 Department of Epidemiology and Biostatistics, Graduate School of Public Health and Health Policy, City University of New York, New York, NY USA; 13 Department of Neurology, University of California, Los Angeles, Los Angeles, CA USA; 14 Department of Computational Medicine, University of California, Los Angeles, Los Angeles, CA USA; 15 Department of Internal Medicine, Henry Ford Health System, Detroit, MI USA; 16 Colorado Center for Personalized Medicine, University of Colorado Anschutz Medical Campus, Aurora, CO USA; 17 Department of Biomedical Informatics, University of Colorado Anschutz Medical Campus, Aurora, CO USA; 18 Helen Diller Family Comprehensive Cancer Center, University of California, San Francisco, San Francisco, CA USA

**Keywords:** Gene expression, Epidemiology, Population genetics

## Abstract

We explored ancestry-related differences in the genetic architecture of whole-blood gene expression using whole-genome and RNA sequencing data from 2,733 African Americans, Puerto Ricans and Mexican Americans. We found that heritability of gene expression significantly increased with greater proportions of African genetic ancestry and decreased with higher proportions of Indigenous American ancestry, reflecting the relationship between heterozygosity and genetic variance. Among heritable protein-coding genes, the prevalence of ancestry-specific expression quantitative trait loci (anc-eQTLs) was 30% in African ancestry and 8% for Indigenous American ancestry segments. Most anc-eQTLs (89%) were driven by population differences in allele frequency. Transcriptome-wide association analyses of multi-ancestry summary statistics for 28 traits identified 79% more gene–trait associations using transcriptome prediction models trained in our admixed population than models trained using data from the Genotype-Tissue Expression project. Our study highlights the importance of measuring gene expression across large and ancestrally diverse populations for enabling new discoveries and reducing disparities.

## Abstract (web summary)

Analyses of whole-genome and RNA sequencing data from 2,733 African American, Puerto Rican and Mexican American individuals reveal ancestry-specific patterns in the genetic architecture of whole-blood gene expression.

## Main

Gene expression has been studied extensively as a trait affected by genetic variation in humans^1^. Expression quantitative trait loci (eQTLs) have been identified in most genes^2–4^ and extensive analyses across multiple tissues have demonstrated both tissue-specific and shared eQTLs^2^. Genome-wide association studies (GWASs) tend to identify loci that are enriched for eQTLs^5^. Colocalization of eQTLs with GWASs is an important element of identifying causal genes and investigating the biology underlying genetic susceptibility to disease^6^. Transcriptome-wide association studies (TWASs) have also been developed to leverage eQTL data by imputing transcriptomic profiles in external datasets or GWAS summary statistics, and have identified additional trait-associated genes^7,8^.

Despite the discovery of thousands of loci that influence hundreds of complex human traits, the underrepresentation of admixed and non-European ancestry individuals in GWAS^9,10^ and multi-omic studies remains an obstacle for applying these approaches to diverse populations. Gene expression prediction models trained in predominantly European ancestry reference datasets, such as the Genotype-Tissue Expression (GTEx) project^2^, have substantially lower accuracy to predict gene expression levels when applied to populations of non-European ancestry^3,11,12^. The importance of aligning ancestry between training and testing populations is also reflected by the poor cross-population performance of other multi-single nucleotide polymorphism (SNP) prediction models, such as polygenic risk scores^13–15^.

To address this gap, we leveraged whole-genome sequencing (WGS) and RNA sequencing (RNA-seq) data from 2,733 participants from the Genes-Environments and Admixture in Latino Asthmatics (GALA II) study and the Study of African Americans, Asthma, Genes, and Environments (SAGE) to characterize the genetic architecture of whole-blood eQTLs. The diversity within the GALA II/SAGE population enabled us to evaluate how genetic ancestry relates to the heritability of gene expression, and to systematically quantify the prevalence of ancestry-specific eQTLs. Lastly, we developed a powerful set of TWAS models from these datasets to facilitate genetic association analyses in multi-ancestry populations.

## Results

**Fig. 1:**
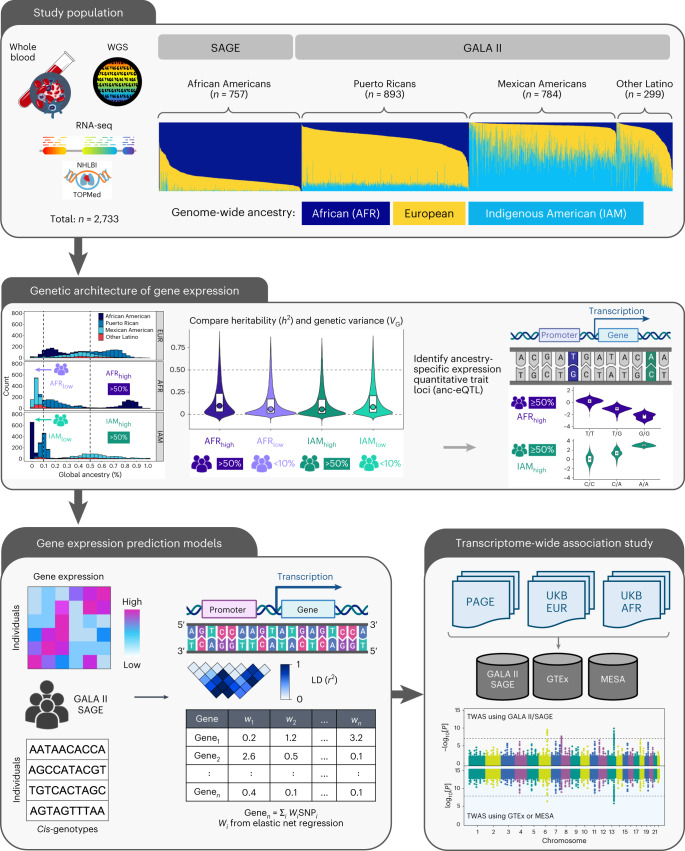
Study overview. This study included TOPMed WGS and whole transcriptome data generated from whole-blood samples of SAGE AA and GALA II Latino individuals (*n* = 2,733). We compared elements of the genetic architecture gene expression, such as *cis*-heritability and genetic variance, across participant groups defined based on self-identified race/ethnicity and genetic ancestry. We performed eQTL mapping and identified eQTLs that were specific to AFR or IAM ancestry. Finally, we developed genetic prediction models of whole-blood transcriptomes and performed comparative TWASs using GWAS summary statistics generated from the PAGE study and the UKB. Figure created with BioRender.com.

We analyzed data from a total of 2,733 participants from the GALA II^16^ and SAGE^17^ asthma case–control studies who self-identified as African American (AA; *n* = 757), Puerto Rican (PR; *n* = 893), Mexican American (MX; *n* = 784) or other Latino American (LA; *n* = 299) (Table 1 and Supplementary Table 1). The median age of the participants varied from 13.2 (PR) to 16.0 years (AA). Genome-wide, or global, genetic ancestry proportions were estimated for all participants (Fig. 1). The median global African ancestry was highest in AA (82.6%), followed by PR (19.7%), and lowest in MX (3.5%).

**Table 1:** Study participants

	Self-identified race/ethnicity								Pooled	
	AA		PR		MX		LA			
Sex										
Number (%) female	405 (53.5)		451 (50.5)		427 (54.5)		158 (52.8)		1,441 (52.7)	
Asthma status										
Number (%) of cases	433 (57.2)		549 (61.5)		351 (44.8)		156 (52.2)		1,489 (54.5)	
Recruitment center (number (%))										
San Francisco Bay Area	757 (100)		0 (0)		348 (44.4)		109 (36.5)		1,214 (44.4)	
Chicago	0 (0)		31 (3.5)		247 (31.5)		52 (17.4)		330 (12.1)	
Puerto Rico	0 (0)		837 (93.7)		0 (0)		8 (2.7)		845 (30.9)	
New York City	0 (0)		22 (2.5)		36 (4.6)		86 (28.8)		144 (5.3)	
Houston	0 (0)		3 (0.3)		153 (19.5)		44 (14.7)		200 (7.3)	
Median (IQR) age (years)	16.0 (6.6)		13.2 (4.8)		13.8 (6.5)		13.7 (5.7)		14.0 (6.3)	
Median (IQR) genetic ancestry (%)										
African	82.6 (9.4)		19.7 (13.3)		3.5 (2.7)		8.3 (14.8)		17.5 (61.8)	
Indigenous American	0.3 (0.9)		9.9 (3.6)		55.3 (23.2)		42.3 (43.2)		10.7 (45.2)	
European	16.5 (9.5)		69.5 (13.6)		40.3 (21.9)		45.9 (20.8)		44.2 (43.8)	
Total	757		893		784		299		2,733	

Demographic characteristics of 2,733 participants from GALA II and SAGE included in the present analysis.

IQR, interquartile range.

### Heritability of gene expression in admixed populations

**Fig. 2:**
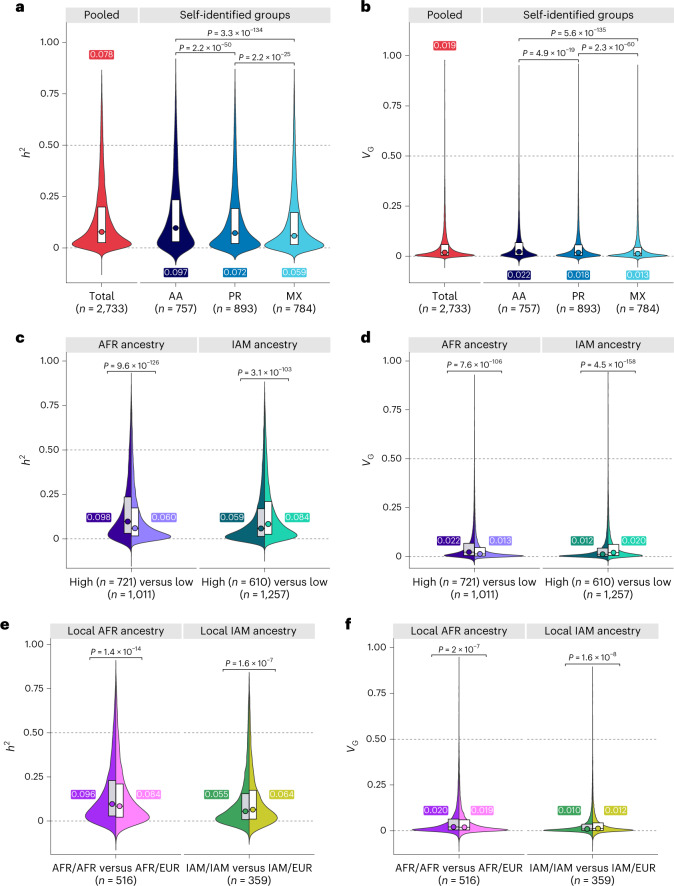
Cross-population comparison of *cis*-heritability (*h*^2^) and genetic variance (*V*_G_) of whole-blood transcript levels. **a**,**b**, Violin plots showing the distribution of *h*^2^ (**a**) and *V*_G_ (**b**) for all available genes in each population. The box plots extend from the 25th to 75th percentiles and median values are annotated. The analyses were stratified by self-identified race/ethnicity and compared AA, PR and MX participants. **c**,**d**, Split violin plots showing the distribution of *h*^2^ (**c**) and *V*_G_ (**d**) in individuals with >50% global genetic ancestry (high; darker shading) versus those with <10% of the same ancestry (low; lighter shading). Analyses were conducted separately for AFR and IAM ancestry. **e**,**f**, The local ancestry at the transcription start site of each gene was used to compare *h*^2^ (**e**) and *V*_G_ (**f**) in participants with 100% (AFR/AFR or IAM/IAM; darker shading) versus 50% (AFR/EUR or IAM/EUR; lighter shading) local ancestry. Statistical significance in all panels was determined by Wilcoxon test and all *P* values are two sided.

**Extended Data Fig. 1:**
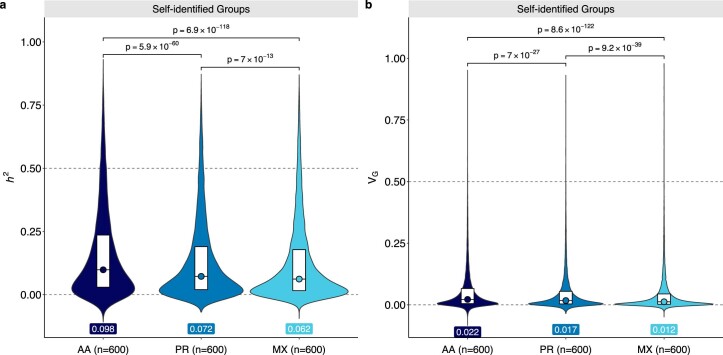
Comparison of *cis*-heritability (*h*^2^) and genetic variance (V_G_) of whole blood transcript levels across populations with a fixed sample size. Analyses of **a**, *h*^2^ and **b**, V_G_ were stratified by self-identified race/ethnicity and compared African American (AA), Puerto Rican (PR), Mexican American (MX) participants. Within each self-identified race/ethnicity group, individuals were down-sampled to n = 600. Violin plots show the full distribution of h^2^ or V_G_ values across all genes within each population. Box plots extend from the 25^th^ to the 75^th^ percentile. Each group-specific median value of h^2^ or V_G_ is annotated and indicated by the shaded circle. All Wilcoxon p-values are two-sided.

We compared the heritability (*h*^2^) and genetic variance (*V*_G_) of whole-blood gene expression attributed to common genetic variation (minor allele frequency (MAF) ≥ 0.01) within the *cis*-region across self-identified race/ethnicity groups and subpopulations defined based on genetic ancestry (see Methods). Across 17,657 genes, *cis*-heritability (Fig. 2a) was significantly higher in AA (median *h*^2^ = 0.097) compared with PR (*h*^2^ = 0.072; *P*_Wilcoxon_ = 2.2 × 10^−50^) and MX participants (*h*^2^ = 0.059; *P* = 3.3 × 10^−134^) and in PR compared with MX participants (*P*_Wilcoxon_ = 2.2 × 10^−25^) (Supplementary Table 2). Genetic variance (Fig. 2b) of whole-blood transcript levels in AA participants (median *V*_G_ = 0.022) was higher than in PR participants (*V*_G_ = 0.018; *P*_Wilcoxon_ = 4.0 × 10^−19^) and in MX participants (*V*_G_ = 0.013; *P*_Wilcoxon_ = 5.6 × 10^−135^). The results remained unchanged when the sample size was fixed to *n* = 600 in all populations (Extended Data Fig. 1).

**Extended Data Fig. 2:**
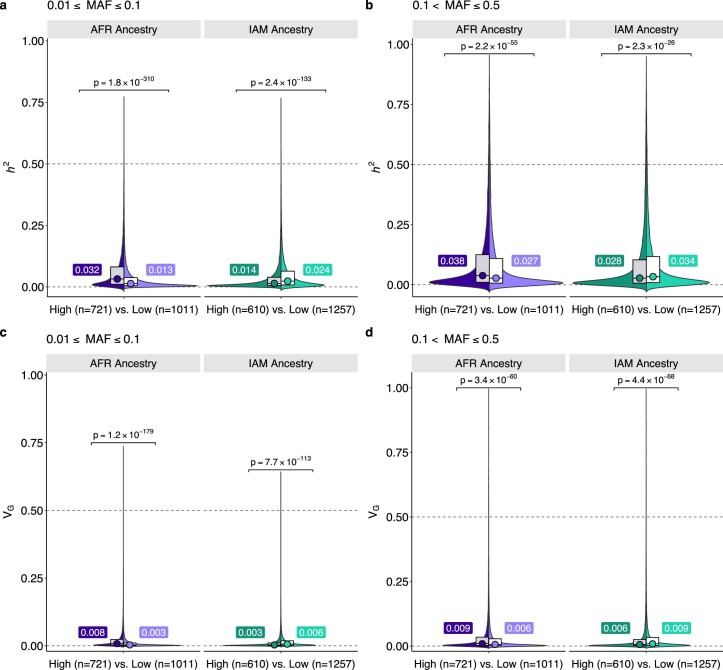
Comparison of *cis*-heritability (*h*^2^) and genetic variance (V_G_) of whole blood transcript levels stratified by minor allele frequency (MAF). Analyses were performed separately for low-frequency variants with 0.01 ≤MAF ≤ 0.1 and for common variants with 0.01 ≤MAF < 0.5. Split violin plots show the distribution of **a, b**, *h*^2^ and **c, d**, V_G_ in individuals with >50% global genetic ancestry (High) to participants with <10% of the same ancestry (Low). Analyses were conducted separately for African (AFR) and Indigenous American (IAM) ancestry within each MAF bin. Box plots extend from the 25^th^ to the 75^th^ percentile and the median value for h^2^ and V_G_ value is annotated. All Wilcoxon p-values are two-sided.

Next, we compared the distribution of *h*^2^ (Fig. 2c) and *V*_G_ (Fig. 2d) between participants grouped based on proportions of global genetic ancestry (Supplementary Table 3 and Supplementary Fig. 1). Among participants with >50% African ancestry (AFR_high_; *n* = 721), *cis-*heritability (*h*^2^ = 0.098) and genetic variance (*V*_G_ = 0.022) were higher than in *n* = 1,011 participants with <10% global African ancestry (AFR_low_; *h*^2^ = 0.060 (*P*_Wilcoxon_ = 9.6 × 10^−126^) and *V*_G_ = 0.013 (*P*_Wilcoxon_ = 7.6 × 10^−106^)). Among individuals with >50% Indigenous American (IAM) ancestry (IAM_high_; *n* = 610), *cis*-heritability (*h*^2^ = 0.059) and genetic variance (*V*_G_ = 0.012) were lower than in participants with <10% IAM ancestry (IAM_low_; *h*^2^ = 0.084 (*P* = 3.1 × 10^−103^) and *V*_G_ = 0.020 (*P*_Wilcoxon_ = 3.1 × 10^−158^)). The results remained consistent when partitioning *h*^2^ and *V*_G_ by coarse MAF bins, with larger differences in *h*^2^ and *V*_G_ among 0.01 ≤ MAF ≤ 0.10 variants (Extended Data Fig. 2).

We also investigated the impact of ancestry at the locus level, defined as the number of alleles (zero, one or two) derived from each ancestral population at the transcription start site. Heritability was higher in individuals with homozygous local African ancestry (AFR/AFR) compared with AFR/European (EUR) ancestry (*h*^2^ = 0.096 versus *h*^2^ = 0.084, respectively; *P*_Wilcoxon_ = 1.4 × 10^−14^) and lower in participants with homozygous Indigenous American ancestry (IAM/IAM) compared with IAM/EUR ancestry (*h*^2^ = 0.055 versus 0.064, respectively; *P* = 1.6 × 10^−7^) (Fig. 2e). Differences in *V*_G_ by local ancestry were statistically significant for AFR/AFR versus AFR/EUR (*P*_Wilcoxon_ = 2.0 × 10^−7^) and IAM/IAM versus IAM/EUR (*P*_Wilcoxon_ = 1.6 × 10^−8^) (Fig. 2f). The results were also consistent for *V*_G_ comparisons within self-identified race/ethnicity groups (Supplementary Table 4).

We calculated heritability using linkage disequilibrium adjusted kinships (LDAKs). This method assumes that SNP-specific variance is inversely proportional not only to the MAF, but also to linkage disequilibrium tagging^18^. Estimates obtained using the LDAK-Thin model and genome-wide complex trait analysis (GCTA) were nearly identical across populations based on self-identified race/ethnicity (*h*^2^ = 0.094 for AA, 0.071 for PR and 0.059 for MX) and genetic ancestry (*h*^2^ = 0.104 for AFR_high_, 0.066 for AFR_low_, 0.062 for IAM_high_ and 0.093 for IAM_low_; Supplementary Table 5).

Lastly, we tabulated the number of heritable genes for which global and/or local ancestry was significantly associated (false discovery rate (FDR) < 0.05) with transcript levels (Supplementary Fig. 2 and Supplementary Table 6). Global AFR ancestry was associated with the expression of 326 (2.4%) and 589 (4.5%) heritable genes in AA and PR, respectively. Associations with local, but not global, AFR ancestry were more common (8.9% in AA and 10.9% in PR). Local IAM ancestry was associated with the expression of 9.8% of genes in MX, compared with 2.8% for global IAM ancestry.

### Assessment of ancestry-specific eQTLs

**Extended Data Fig. 3:**
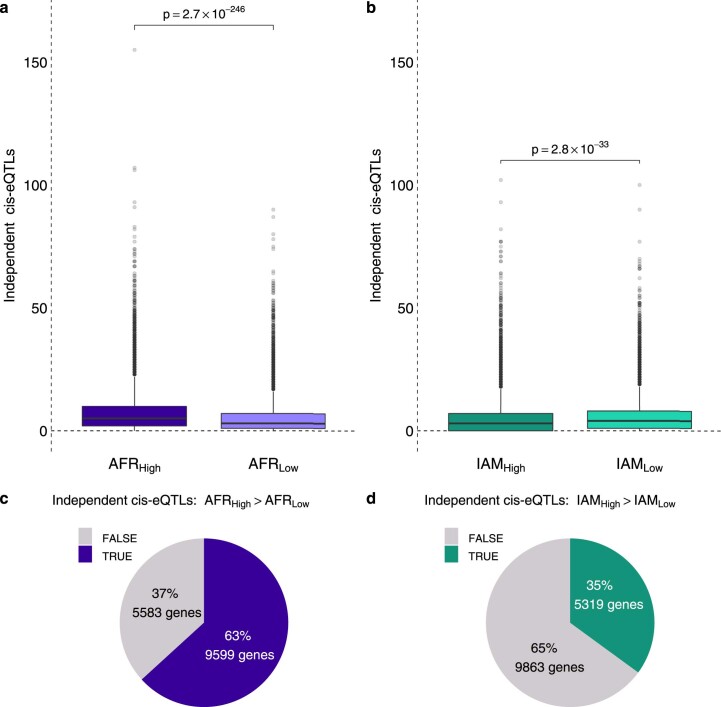
Cross-population comparison of independent cis-eQTLs. Sample size was fixed to n = 600 for eQTL mapping analyses in each ancestry group. Independent cis-eQTLs were identified by performing LD-based clumping (*r*^2^ < 0.10) of statistically significant eQTLs identified in each population. Individuals with >50% global genetic ancestry (High) were compared to participants with <10% of the same ancestry (Low). Analyses were conducted separately for African (AFR) and Indigenous American (IAM) ancestry. The distribution of independent cis-eQTLs per gene between was compared between **a**, AFR_high_ and AFR_low_ populations and **b**, IAM_high_ and IAM_low_ groups using a two-sided Wilcoxon test. Pie charts visualize the proportion of genes with a greater number of cis-eQTLs in **c**, AFR_high_ compared to AFR_low_ and in **d**, IAM_high_ compared to IAM_low_.

To understand the control of gene expression at a more granular level, we performed *cis*-eQTL analysis. A total of 19,567 genes with at least one *cis*-eQTL (eGenes) were found in the pooled sample. The largest number of eGenes was detected in AA (*n* = 17,336), followed by PR (*n* = 16,975) and MX participants (*n* = 15,938) (Supplementary Table 7 and Supplementary Fig. 3). The number of eGenes was similar in AFR_high_ (*n* = 17,123) and AFR_low_ (*n* = 17,146) groups. When the sample size was fixed to *n* = 600 for all populations, the number of eGenes observed in AFR_high_ (*n* = 16,100) was higher than in AFR_low_ (*n* = 14,344). The numbers of eGenes detected in the IAM_low_ (*n* = 14,866) and IAM_high_ groups (*n* = 14,419) were similar. The number of linkage disequilibrium-independent (*r*^2^ < 0.10) *cis*-eQTLs per gene was significantly higher in the AFR_high_ group than the AFR_low_ group (*P*_Wilcoxon_ = 2.7 × 10^−246^) and lower in the IAM_high_ group compared with the IAM_low_ group (*P*_Wilcoxon_ = 2.8 × 10^−33^) (Extended Data Fig. 3).

**Fig. 3:**
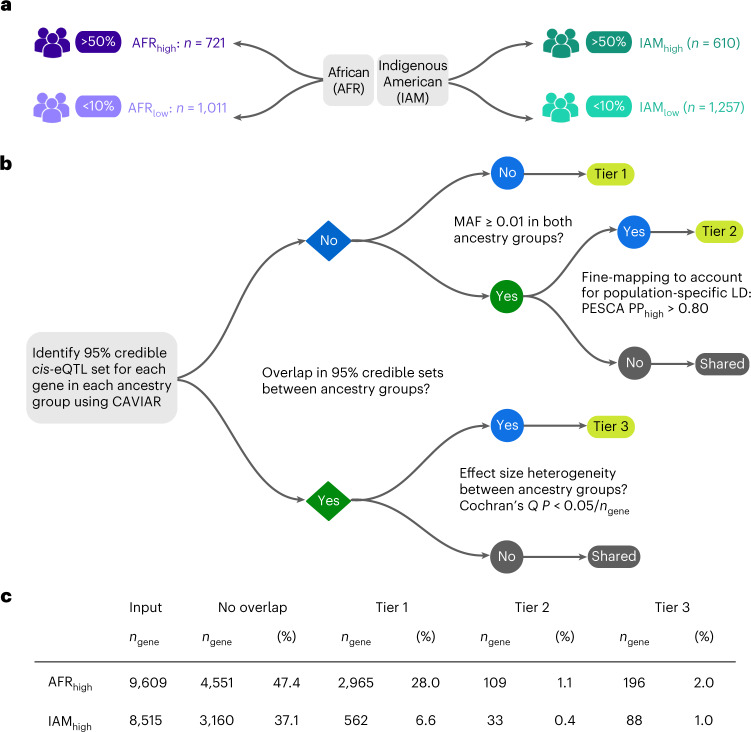
Framework for the classification of anc-eQTLs. **a**, eQTL mapping analyses were conducted in participant groups derived based on genetic ancestry. Associations identified in participants with >50% global African ancestry (AFR_high_; *n* = 721) were compared with eQTLs detected in participants with <10% African ancestry (AFR_low_; *n* = 1,011), and eQTLs identified in participants with >50% global Indigenous American ancestry (IAM_high_; *n* = 610) were compared with associations detected in participants with <10% Indigenous American ancestry (IAM_low_; *n* = 1,257). **b**, Decision tree for the classification of anc-eQTLs. Tier 1 is based on MAF differences and represents the most ancestry-specific class. Tier 2 anc-eQTLs were identified using additional fine-mapping with PESCA^22^. Tier 3 anc-eQTLs were detected in both ancestry groups, but with statistically significant effect size heterogeneity based on Cochran’s *Q* test. **c**, Prevalence of anc-eQTLs in each ancestry group. Figure created with BioRender.com.

To characterize ancestry-related differences in the regulation of gene expression, we developed a framework for identifying ancestry-specific eQTLs (anc-eQTLs) (see Methods, Fig. 3 and Supplementary Tables 8 and 9). For heritable protein-coding genes, we first compared the overlap in 95% credible sets of *cis*-eQTLs identified in participants with >50% global ancestry (AFR_high_ and IAM_high_) and those with <10% of the same global ancestry (AFR_low_ and IAM_low_). For genes with nonoverlapping 95% credible sets, we distinguished between population differences in MAF (tier 1) and linkage disequilibrium (tier 2). For genes with overlapping 95% credible sets, eQTLs were further examined for evidence of effect size heterogeneity between ancestry groups (tier 3).

**Fig. 4:**
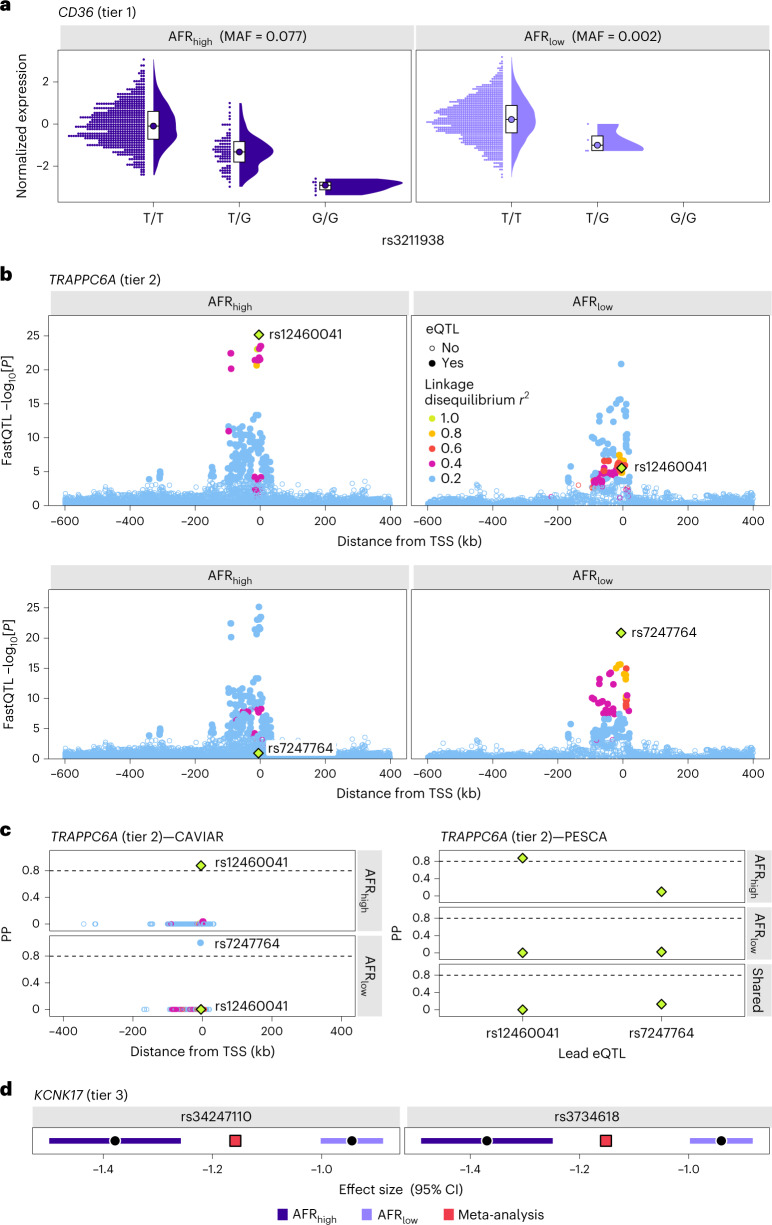
Examples of anc-eQTLs. **a**, The tier 1 anc-eQTL (rs3211938) for *CD36* is present in individuals with >50% African ancestry (AFR_high_) with an MAF of 0.077 and in individuals with <10% African ancestry (AFR_low_) with an MAF of 0.002. *CD36* expression by rs3211938 genotype is shown for each individual and summarized using split violin plots and box plots, which extend from the 25th to the 75th percentile. **b**, Regional plots of FastQTL association results for *TRAPPC6A* show two independent eQTL signals in AFR_high_ and AFR_low_ participants, with linkage disequilibrium *r*^2^ < 0.2 in each population. Variants are colored based on linkage disequilibrium *r*^2^ with respect to the index variant, as indicated by the diamond. Hollow circles show variants that were not associated with *TRAPPC6A* expression at the gene-specific *P* value threshold. kb, kilobases; TSS, transcription start site. **c**, Left, fine-mapping using CAVIAR identified nonoverlapping 95% credible sets in AFR_high_ and AFR_low_ groups. The lead AFR_high_ eQTL, rs12460041, had a PP of 0 in AFR_low_. Right, fine-mapping using PESCA to account for linkage disequilibrium differences between populations confirmed that rs12460041 is a tier 2 anc-eQTL with a PP of >0.80 in AFR_high_. **d**, Tier 3 anc-eQTLs rs34247110 and rs3734618 were included in the 95% credible set for *KCNK17* in AFR_high_ and AFR_low_ groups, but have different eQTL effect sizes in each population, as indicated by the nonoverlapping shaded 95% CIs.

Tier 1 anc-eQTLs were only common (MAF ≥ 0.01) in individuals with >50% AFR or IAM ancestry and were thus considered to be the most ancestry specific. Over 28% (*n* = 2,695) of genes contained at least one tier 1 AFR_high_ anc-eQTL, while 7% (*n* = 562) of genes contained a tier 1 IAM_high_ anc-eQTL (Supplementary Table 9). A representative example of a tier 1 AFR_high_ anc-eQTL is rs3211938 (*CD36*), which has an MAF of 0.077 in the AFR_high_ group and an MAF of 0.002 in the AFR_low_ group (Fig. 4a). This variant has been linked to high-density lipoprotein (HDL) cholesterol levels in several multi-ancestry GWASs that included African Americans^19–21^. Tier 2 anc-eQTLs, with ancestry-specific linkage disequilibrium patterning, had an MAF of 0.01 in both high (>50%) and low (<10%) global ancestry groups and were further fine-mapped using PESCA^22^. There were 109 genes (1.1%) that contained eQTLs with a posterior probability (PP) of being specific to AFR_high_ of >0.80 and 33 genes (0.4%) matching the same criteria for IAM_high_ (Supplementary Table 9). For instance, two lead eQTLs in low linkage disequilibrium were detected for *TRAPPC6A* in AFR_high_ (rs12460041) and AFR_low_ (rs7247764) populations (Fig. 4b). These variants belonged to nonoverlapping credible sets and PESCA estimated that rs12460041 was specific to AFR_high_ with a PP of 0.87 (Fig. 4c). Over 50% of heritable protein-coding genes (*n* = 5,058 for AFR and 5,355 for IAM) had overlapping 95% credible sets of eQTLs between high and low ancestry groups. Among these shared signals, a small proportion of eQTLs exhibited significant effect size heterogeneity (tier 3; 2.0% for AFR_high_ and 1.0% for IAM_high_). For example, the *KCNK17* eQTLs rs34247110 and rs3734618 were detected in the AFR_high_ and AFR_low_ groups, but with significantly different effect sizes (Cochran’s *Q* *P* value = 1.8 × 10^−10^) in each population (Fig. 4d). Consistent with tier 3 eQTLs being observed in multiple ancestries, rs34247110 has been associated with type 2 diabetes in Japanese and multi-ancestry (European, African American, Hispanic and Asian) populations^23,24^.

**Extended Data Fig. 4:**
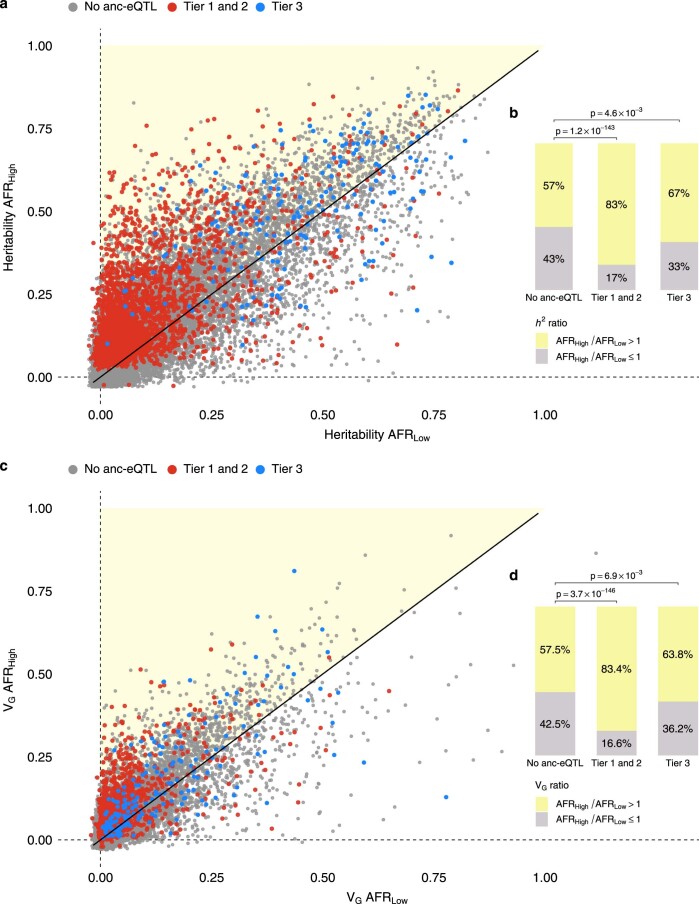
Scatter plots comparing gene-specific *h*^*2*^ and V_G_ by levels of African ancestry. Estimates of **a**, h^2^ and **c**, V_G_ for each gene in individuals with ≥50% global African ancestry (AFR_High_) are plotted on the y-axis and in participants with <10% AFR ancestry (AFR_Low_) on the x-axis. Genes containing ancestry-specific eQTLs are highlighted. The proportion of genes falling off the diagonal, with higher **b**, h^2^ or **d**, V_G_ in AFR_High_ than AFR_Low_, is show using stacked bar plots and compared using a two-sided binomial test.

The prevalence of any tier 1, 2 or 3 anc-eQTL was 30% (*n* = 2,961) for AFR ancestry and 8% (*n* = 679) for IAM ancestry. Overall, 3,333 genes had anc-eQTLs for either ancestry. The remaining genes (*n* = 6,648 for AFR and 7,836 for IAM) did not contain eQTLs with ancestry-related differences in MAF, linkage disequilibrium or effect size as outlined above. Increasing the global ancestry cut-off to >70% did not have an appreciable impact on anc-eQTLs in the AFR_high_ group (28.1% overall and 27.3% for tier 1), but decreased the number of anc-eQTLs in the IAM_high_ group (3.3% overall and 3.3% for tier 1), probably due to a greater reduction in sample size in this group (*n* = 212 versus *n* = 610, respectively; Supplementary Table 10). Considering all protein-coding genes without filtering based on heritability (*n* = 13,535), the prevalence of anc-eQTLs was 22% for the AFR_high_ group, 5% for the IAM_high_ group and 25% overall. The observation that anc-eQTLs were more common in participants with >50% AFR ancestry aligns with the higher *h*^2^ and *V*_G_ values in this population and a greater number of linkage disequilibrium-independent *cis*-eQTLs (Extended Data Fig. 3). Among genes with tier 1 and 2 anc-eQTLs, 83% had higher *h*^2^ estimates in the AFR_high_ group than in the AFR_low_ group, while this was observed for 57% of genes without any ancestry-specific eQTLs (Extended Data Fig. 4).

**Extended Data Fig. 5:**
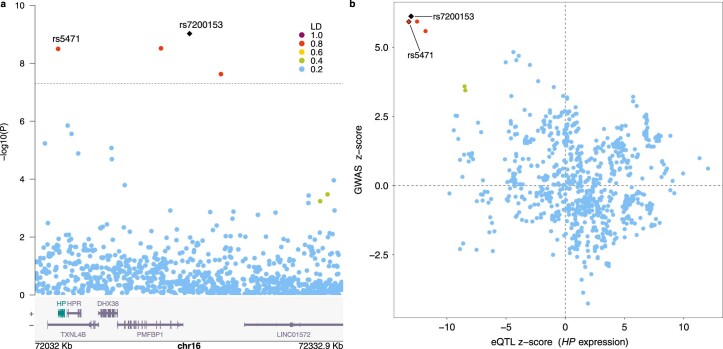
Colocalization of haptoglobin (*HP*) expression and total cholesterol. We observed strong evidence of colocalization, with posterior probability (PP) = 0.997, between Tier 1 ancestry-specific eQTLs for *HP* and GWAS associations from PAGE for total cholesterol. **a**, Regional plot showing GWAS results for total cholesterol in PAGE. The labeled eQTLs comprise the 95% credible set for *HP*: rs7200153 (PP_SNP_ = 0.519) and rs5471 (PP_SNP_ = 0.481). Variants are colored based on LD with respect to rs7200153, which had the lowest GWAS p-value in PAGE. **b**, Plot of test statistics for variant effects on gene expression (eQTL z-scores) against effects on total cholesterol observed in PAGE (GWAS z-scores).

We detected 70 unique anc-eQTLs associated with 84 phenotypes from the NHGRI-EBI GWAS catalog^25^, most of which were tier 3 anc-eQTLs (59%) that mapped to blood cell traits, lipids and plasma protein levels (Supplementary Table 11). Colocalization with GWAS results from the multi-ancestry Population Architecture Using Genomics and Epidemiology (PAGE) study^21^ identified 78 eQTL–trait pairs with strong evidence of a shared genetic signal (PP_4_ > 0.80), 16 of which were anc-eQTLs (Supplementary Table 12). One illustrative example is rs7200153, an AFR_high_ tier 1 anc-eQTL for the haptoglobin (*HP*) gene, which colocalized with total cholesterol (PP_4_ = 0.997; Extended Data Fig. 5). The 95% credible set included rs7200153 (PP_SNP_ = 0.519) and rs5471 (PP_SNP_ = 0.481; linkage disequilibrium *r*^2^ = 0.75), with rs5471 probably being the true causal variant given its proximity to the *HP* promoter, stronger effect of *HP* expression and decreased transcriptional activity of rs5471-C in West African populations^26–28^, which is supported by previous literature^20,29,30^.

We also performed *trans*-eQTL analyses that largely recapitulated our *cis*-eQTL results, with a larger number of *trans*-eGenes and independent (linkage disequilibrium *r*^2^ < 0.10) *trans*-eQTLs in populations with greater levels of AFR ancestry and lower levels of IAM ancestry (see Methods and Supplementary Table 13).

### Performance of TWAS models trained in admixed populations

Following the PrediXcan approach^7^, we developed gene expression imputation models from the pooled GALA II and SAGE population (*n* = 2,733) for 11,830 heritable genes with a mean cross-validation of 0.157 (Supplementary Table 13 and Supplementary Fig. 4). We also generated population-specific models for African Americans (10,090 genes; cross-validation *r*^2^ = 0.180), Puerto Ricans (9,611 genes; cross-validation *r*^2^ = 0.163) and Mexican Americans (9,084 genes; cross-validation *r*^2^ = 0.167). Adjusting for local ancestry did not improve predictive performance (Supplementary Table 14).

We validated GALA II/SAGE TWAS models and compared with GTEx v8 in the Study of Asthma Phenotypes and Pharmacogenomic Interactions by Race-Ethnicity (SAPPHIRE)^31^—a study of 598 African American adults (Supplementary Fig. 5). The prediction accuracy was proportional to the degree of alignment in ancestry between the training and testing study samples. Across 5,254 genes with models available in all studies, the median Pearson’s correlation between genetically predicted and observed transcript levels was highest for pooled (*r* = 0.086) and AA (*r* = 0.083) models and lowest for GTEx (*r* = 0.049).

**Fig. 5:**
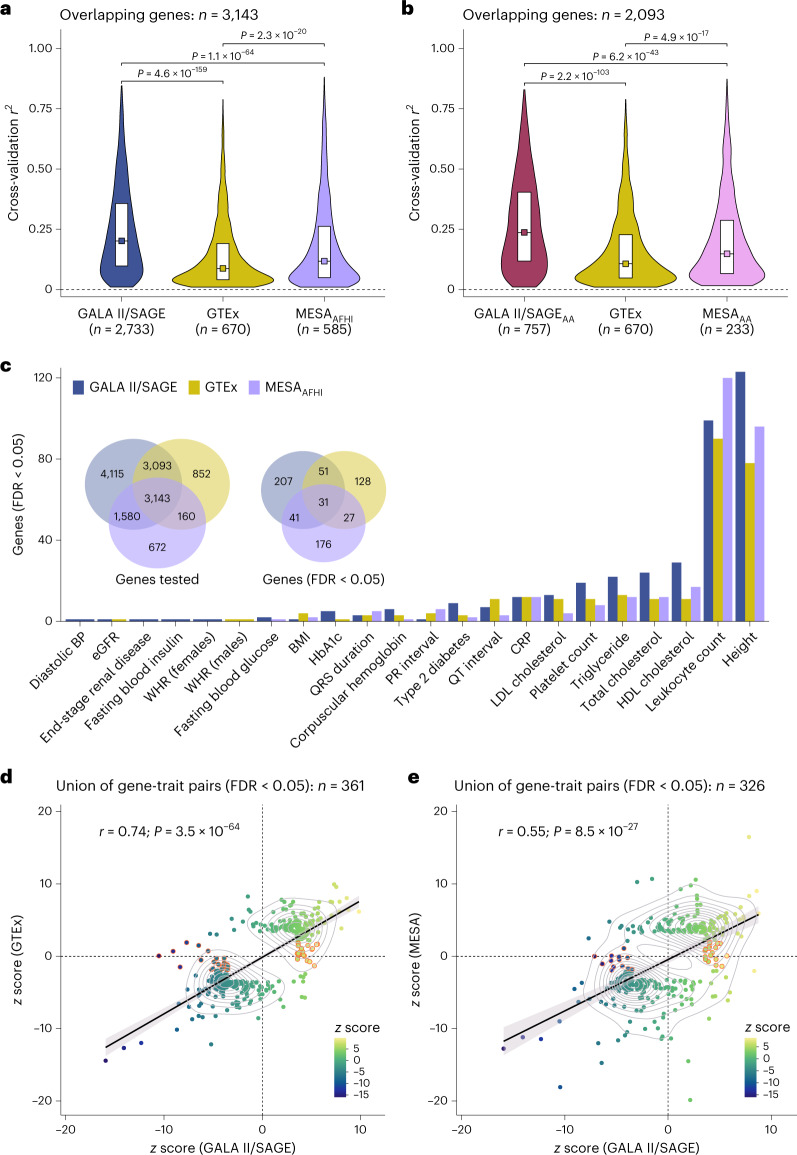
Comparison of transcriptome imputation model performance and TWAS results. TWAS models were developed using data from GALA II and SAGE participants following the PredictDB pipeline and compared with TWAS models generated in GTEx v8 and MESA using the same approach. **a**,**b**, Violin plots showing the distribution of internal cross-validation *r*^2^ values for genes with TWAS models available in each study. The box plots extend from the 25th to the 75th percentiles of the *r*^2^ distributions, with median *r*^2^ values indicated by squares. Predictive performance was compared between TWAS models trained in the pooled GALA II/SAGE population and MESA models trained in African American and Hispanic participants (MESA_AFHI_) (**a**) and between TWAS models trained in African Americans (**b**). Statistical significance was determined by Wilcoxon test and the *P* values for differences in *r*^2^ are two sided. **c**, Summary of TWAS associations with FDR < 0.05 across 28 traits in PAGE. BMI, body mass index; BP, blood pressure; CRP, C-reactive protein; eGFR, estimated glomerular filtration rate; HbA1c, hemoglobin A1c; LDL, low-density lipoprotein; WHR, waist-to-hip ratio. **d**,**e**, Correlation between TWAS *z* scores based on GALA II/SAGE pooled models and *z* scores using GTEx (**d**) or MESA_AFHI_ models (**e**) for the union of genes with FDR < 0.05 detected with either model. Associations between TWAS *z* scores from each model are visualized by linear regression lines with shaded 95% CIs. Genes highlighted in orange had FDR < 0.05 using GALA II/SAGE models but did not reach nominal significance (TWAS *P* value > 0.05) using GTEx or MESA models.

To evaluate the performance of the GALA II/SAGE TWAS models for gene discovery in admixed populations, we applied them to GWAS summary statistics for 28 traits from the PAGE study^21^ and compared them with TWASs using GTEx v8 (refs. ^2,7^) and the Multi-Ethnic Study of Atherosclerosis (MESA)^3^. GTEx v8 whole-blood models are based on 670 participants of predominantly European ancestry (85%)^2^, while MESA models impute monocyte gene expression^3^ from African American and Hispanic/Latino individuals (MESA_AFHI_; *n* = 585). The number of genes with available TWAS models was 39–82% higher in GALA II/SAGE compared with GTEx (*n* = 7,249) and MESA_AFHI_ (*n* = 5,555). Restricting to 3,143 genes shared across all three studies, the cross-validation *r*^2^ was significantly higher in GALA II/SAGE compared with GTEx (*P*_Wilcoxon_ = 4.6 × 10^−159^) and MESA_AFHI_ (*P*_Wilcoxon_ = 1.1 × 10^−64^) (Fig. 5a). TWAS models generated in GALA II/SAGE AA (*n* = 757) attained a higher cross-validation *r*^2^ than GTEx (*P*_Wilcoxon_ = 2.2 × 10^−103^), which had a comparable training sample size (Fig. 5b).

TWASs using GALA II/SAGE models across 28 PAGE traits identified a larger number of significant gene–trait pairs (*n* = 380; FDR < 0.05) than MESA_AFHI_ (*n* = 303) and GTEx (*n* = 268), with only 35 significant gene–trait pairs detected in all three analyses (Fig. 5c). GALA II/SAGE models yielded a larger number of associated genes than MESA in 80% of analyses (binomial test; *P* = 0.012) and a larger number than GTEx in 79% of analyses (binomial test; *P* = 0.019). Of the 330 genes with an FDR of <0.05 in GALA II/SAGE, 143 (43%) were not present in GTEx and 199 (60%) were not present in MESA_AFHI_. For genes that were significant in at least one TWAS, *z* scores in GALA II/SAGE were highly correlated with GTEx (*r* = 0.74; *P* = 3.5 × 10^−64^; Fig. 5c) and MESA_AFHI_ (*r* = 0.55; *P* = 8.5 × 10^−27^; Fig. 5d), suggesting that most genes have concordant effects even if they are not significant in both analyses.

**Fig. 6:**
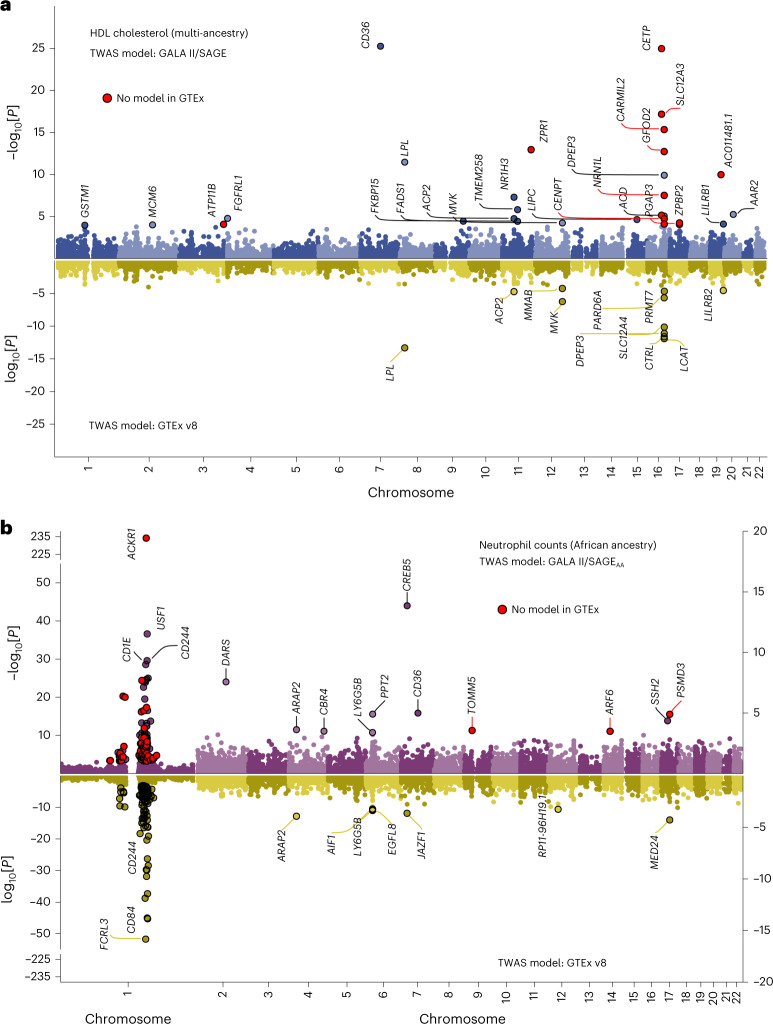
Comparison of TWAS results for selected traits. **a**, A TWAS of HDL was performed by applying GALA II/SAGE pooled models and GTEx v8 models to GWAS summary statistics from the multi-ancestry PAGE study (*n* = 33,063). **b**, A TWAS of neutrophil counts was performed by applying GALA II/SAGE models trained in African Americans and GTEx v8 to summary statistics from a GWAS meta-analysis of individuals of African ancestry (*n* = 13,476) by Chen et al.^32^. All genes with FDR < 0.05 are labeled, except for chromosome 1 in **b** due to the large number of statistically significant associations. Significantly associated genes for which expression levels could not be predicted using GTEx v8 elastic net models are highlighted in red.

HDL cholesterol exhibited one of the largest differences in TWAS associations (Fig. 6a), with over 60% more significant genes identified using GALA II/SAGE models (*n* = 29) than GTEx predictions (*n* = 11). TWAS models for several associated genes, including those with established effects on cholesterol transport and metabolism, such as *CETP*, were not available in GTEx. The top HDL-associated gene, *CD36* (*z* score = −10.52; *P*_TWAS_ = 6.9 × 10^−26^) had tier 1 AFR_high_ anc-eQTLs (rs3211938) that were rare in European ancestry populations (MAF = 0.00013). The difference in MAF may explain why *CD36* was not detected using GTEx (*z* score = 0.057; *P*_TWAS_ = 0.95), even though all 43 variants from the GTEx model were available in PAGE summary statistics.

Although GALA II/SAGE multi-ancestry TWAS models showed robust performance, in some cases population-specific models may be preferred. For instance, benign neutropenia is a well-described phenomenon in persons of African ancestry and is attributed to variation in the 1q23.2 region. Applying GALA II/SAGE AA models to a meta-analysis of 13,476 individuals of African ancestry^32^ identified *ACKR1* (*P*_TWAS_ = 1.5 × 10^−234^), the atypical chemokine receptor gene that is the basis of the Duffy blood group system (Fig. 6b). This causal gene was missed by GTEx and MESA_AFA_. After conditioning on the Duffy-null rs2814778-CC genotype, no statistically significant TWAS associations remained on chromosome 1 (Supplementary Fig. 6). GALA II/SAGE AA models also detected seven genes outside of 1q23.2 that were not previously reported in GWASs of neutrophil counts: *CREB5* (*P*_TWAS_ = 1.5 × 10^−14^), *DARS* (*P*_TWAS_ = 2.9 × 10^−8^), *CD36* (*P*_TWAS_ = 1.1 × 10^−5^), *PPT2* (*P*_TWAS_ = 1.3 × 10^−5^), *SSH2* (*P*_TWAS_ = 4.7 × 10^−5^), *TOMM5* (*P*_TWAS_ = 2.9 × 10^−4^) and *ARF6* (*P*_TWAS_ = 3.4 × 10^−4^).

**Extended Data Fig. 6:**
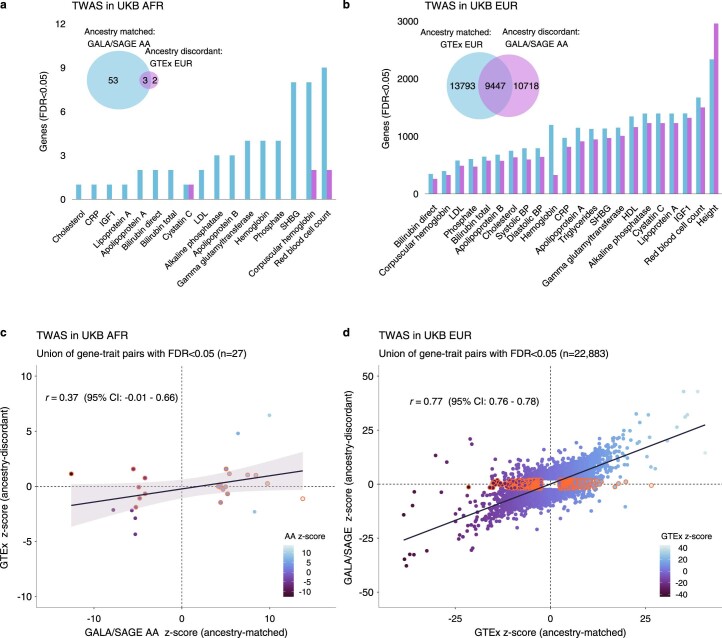
Summary of cross-ancestry TWAS results in UK Biobank. TWAS of 22 blood-based biomarkers and quantitative traits was performed using GWAS summary statistics from African and European ancestry subjects in the UK Biobank. We applied GTEx v8 whole blood models and GALA/SAGE models trained in African Americans (AA) to each set of summary statistics. The number of genes with false discovery rate (FDR) < 0.05 detected in ancestry matched and ancestry discordant analyses is summarized for TWAS of **a**, African (AFR) and **b**, European (EUR) ancestry subjects. Correlation between TWAS z-scores for statistically significant associations detected in **c**, UKB EUR and **d**, UKB AFR are shown for genes that were present in GTEx and GALA/SAGE models. Association between TWAS z-scores from each model is visualized by a linear regression line with shaded 95% confidence intervals. Genes highlighted in orange had FDR < 0.05 using ancestry-matched models but did not reach nominal significance (TWAS p-value>0.05) using ancestry discordant models.

Next, we performed TWASs of 22 blood-based biomarkers and quantitative traits using summary statistics from the UK Biobank (UKB). Ancestry-matched TWASs of UKB AFR (median GWAS *n* = 6,190) identified 56 gene–trait associations (FDR < 0.05), whereas GTEx detected only five genes (Extended Data Fig. 6). TWAS *z* scores for associated genes were modesty correlated (*r* = 0.37; 95% confidence interval (CI) = −0.01–0.66). TWASs in UKB EUR (median GWAS *n* = 400,223) also illustrated the advantage of ancestry-matched analyses, but the difference was less dramatic, with a 15% decrease in the number of genes that reached an FDR of <0.05 using GALA II/SAGE AA models and strong correlation between *z* scores (*r* = 0.77; 95% CI = 0.76–0.78). Concordance between significant associations across all traits was 28%, ranging from 32.7% for height to 7.6% for hemoglobin.

## Discussion

Our comprehensive analysis in a diverse population elucidated the role of genetic ancestry in shaping the genetic architecture of whole-blood gene expression that may be applicable to other complex traits. We found that *cis*-heritability and genetic variance of gene expression increased with a higher proportion of global African ancestry, and that in admixed populations heritability was also highest in individuals with predominantly local African ancestry. We also found that *cis*-*h*^2^ and *V*_G_ were lower in individuals with higher levels of Indigenous American compared with European ancestry. The consistent effects of locus-specific ancestry on *h*^2^ and *V*_G_ within each population suggest that confounding by social or environmental factors is unlikely to explain these results. The relationship between ancestry and heritability that we demonstrated for whole-blood gene expression has not been previously shown in a sufficiently large and diverse population with WGS data.

Our findings are consistent with the pattern of heterozygosity in African and Indigenous American populations. Sub-Saharan African populations have the highest heterozygosity since the ancestors of all other populations passed through a bottleneck during their migration out of Africa^33,34^. Indigenous American populations have passed through additional bottlenecks^35,36^, leading to lower heterozygosity^37^. Therefore, greater genetic variance of gene expression in African ancestry populations may be due to more segregating functional variants in the *cis*-region^38^. This is also supported by the higher number of linkage disequilibrium-independent *cis*-eQTLs, overall and per gene, in AFR_high_ compared with AFR_low_ groups.

Our second major finding was that over 30% of heritable protein-coding genes have ancestry-specific eQTLs, most of which are tier 1 variants that are rare (MAF < 0.01) or nonpolymorphic in another population. The prevalence of these anc-eQTLs remained stable when the global ancestry cut-off was increased from 50 to 70%. These findings align with a recent plasma proteome analysis of the Atherosclerosis Risk in Communities study, which found that nearly 33% of protein QTLs identified in a large sample of African Americans (*n* = 1,871) were nonexistent or rare in the 1000 Genomes EUR population^39^. Tier 2 anc-eQTLs were defined as variants present at an MAF of ≥0.01 in both ancestry groups, but which do not belong to the same gene-specific credible set. This eQTL class was far less common than tier 1 and could arise due to differences in environmental effects on gene expression, gene-by-gene and gene-by-environment interactions or multiple causal variants at the same locus. Among eQTL signals that were shared between ancestry groups, effect size heterogeneity was rare and tier 3 anc-eQTLs were effectively eliminated when AFR_high_ and IAM_high_ were defined using 70% as the global ancestry cut-off. However, comparisons of marginal effect sizes are confounded by differences in sampling error, particularly when there is an imbalance in sample size between populations. Therefore, we may have underestimated ancestry-related heterogeneity in eQTL effects.

Our third major finding was that TWAS models trained in the ancestrally diverse GALA II/SAGE population identified significantly more trait-associated genes than models trained in GTEx and MESA when applied to GWAS results from the multi-ancestry PAGE study. GALA II/SAGE TWAS models benefit from having more similar allele frequency profiles and more accurate modeling of linkage disequilibrium, which is consistent with previous observations^11,12,40^ that ancestry-concordant models improve the power for gene discovery in TWASs. Furthermore, over 40% of significantly associated TWAS genes detected using GALA II/SAGE models were not available in GTEx. Biologically informative associations may be missed by relying exclusively on European ancestry-based TWAS models, such as the top two HDL cholesterol-associated genes (*CETP* in 16q13 and *CD36* in 7q21) with established effects on lipid metabolism^20,41–43^. *CD36* expression was associated with multiple phenotypes and contains tier 1 anc-eQTLs found in individuals with >50% African ancestry, consistent with findings of evolutionary pressures at this locus^44,45^. *CD36* encodes a transmembrane protein that binds many ligands, including collagen, thrombospondin and long-chain fatty acids^46^. *CD36* also mediates the adherence of erythrocytes infected with *Plasmodium falciparum*, which causes severe malaria^47,48^.

One of the striking examples of ancestry-specific genetic architecture in our TWASs is the Duffy antigen receptor gene (*ACKR1*) on 1q23.2, where rs2814778 is responsible for constitutively lower white blood cell and neutrophil counts in populations of African ancestry^49,50^. The Duffy-null rs2814778-CC genotype confers resistance to *Plasmodium vivax* malaria and is present at 60–80% frequency in African populations, but is extremely rare in European and Asian populations. The expression of *ACKR1* could not be imputed using GTEx or MESA, but this gene was captured by the pooled and African American GALA II/SAGE models and accounted for the TWAS signal for neutrophil counts in chromosome 1. We also identified 11 genes outside of the Duffy locus, including *DARS1* (which modulates reactivity to mosquito antigens^51^) and *TOMM5* (which has been implicated in lipoprotein phospholipase A2 activity^52^).

Analyses in UKB illustrated that while ancestry-matched training and testing populations are clearly optimal, there is some evidence that transcriptome prediction models developed in African Americans may have better cross-population portability than models trained in predominantly European ancestry cohorts. Similar results were observed for proteome-wide models in the Atherosclerosis Risk in Communities study^37^, where predicted *r*^2^ standardized by *cis*-*h*^2^ was higher for AA models applied to EU than for EU modes in AA. Greater genetic diversity of African ancestry populations probably captures a more comprehensive set of genetic predictors, only a fraction of which may be present in populations that underwent additional bottlenecks. These findings highlight the value of genetic prediction models trained in admixed, and in particular African ancestry, populations as a resource for identifying new trait-associated genes.

PAGE TWAS *z* scores were highly correlated across transcriptome models, although the correlation with GALA II/SAGE estimates was higher for GTEx whole blood than MESA_AFHI_, which may partly reflect differences between whole-blood and monocyte transcriptomes in MESA_AFHI_^2^. In addition, GALA II/SAGE and GTEx conducted whole-genome sequencing, whereas MESA models are based on imputed genotype data.

Since the genetic architecture of complex traits may mirror the genetics of gene expression^53^, higher heritability in individuals with at least 50% global African ancestry implies that genetic prediction of complex traits in this population should be at least as accurate, if not more so, compared with in European populations. However, polygenic prediction of complex traits in almost all populations, especially African ancestry, lags substantially behind European ancestry^14^ due to insufficient sample size and underrepresentation in discovery studies. This is not observed in simulation studies or well-powered analyses of diverse cohorts^38,54,55^. The substantial prevalence of ancestry-specific eQTLs driven by allele frequency differences also implies that analytic approaches alone will yield limited improvements in the cross-population portability of genetic prediction models. For instance, fine-mapping methods that account for differential linkage disequilibrium tagging to identify causal variants will recover some deficits in prediction performance but will not compensate for unobserved risk variants. Our results reinforce the conclusion that developing truly generalizable genetic prediction models requires capture of the full spectrum of genetic variation across human populations. As such, studies that engage with and recruit diverse populations across the globe are more likely to identify novel associations of both public health and biological relevance.

Several limitations of our work should be acknowledged. We only examined whole-blood transcriptomes and analyses of ancestry-specific effects should be conducted for other tissues. However, whole blood is one of the most clinically informative and commonly collected samples, and with the high degree of correlation between gene expression in whole blood and other tissues^56^, our observations regarding the eQTL genetic architecture are probably generalizable. Another limitation of our approach is that we assumed that each gene had one causal eQTL locus in our ancestry comparisons, which may underestimate the number of ancestry-specific eQTLs for genes with multiple independent signals. A caveat of the tier 2 anc-eQTL classification is that PESCA relies on having linkage disequilibrium regions that are approximately independent in both populations to estimate the proportion of causal variants. This is a challenge in admixed populations with longer-range linkage disequilibrium and may lead to biased estimates. Lastly, we compared our TWAS models with elastic net TWAS models from GTEx because they were developed using the same analytic pipeline, although TWAS MASHR models predict a larger number of genes using fine-mapped eQTLs^57^.

Consistent with Gay et al.^58^, we observed that local ancestry explains a larger proportion of variance in gene expression corrected for global ancestry. However, adjustment for local ancestry as a covariate did not improve the predictive performance of our TWAS models. This may be due to overadjustment, as local ancestry may serve as a proxy for information already captured by population-specific genetic variants, or because of how local ancestry was modeled in our analyses.

Despite these limitations, our study leveraged a uniquely large and diverse sample of 2,733 African American, Puerto Rican and Mexican American participants to explore the interplay between genetic ancestry and the regulation of gene expression. Our cross-ancestry analysis of eQTLs serves as a resource for investigators performing colocalization analyses in genetic association studies of populations of African and Indigenous American ancestry. In addition, we provide genetic prediction models of whole-blood transcriptomes that cover a greater number of genes and facilitate more powerful TWASs when applied to studies of admixed individuals and multi-ancestry GWAS meta-analyses. In summary, our study highlights the need for larger genomic studies in globally representative populations for characterizing the genetic basis of complex traits and ensuring equitable translation of precision medicine efforts.

## Methods

### Study population

This study examined African American, Puerto Rican and Mexican American children between 8 and 21 years of age with or without physician-diagnosed asthma from the GALA II study and SAGE. The inclusion and exclusion criteria have previously been described in detail^16,17^. Briefly, participants were eligible if they were 8–21 years of age and identified all four grandparents as Latino for GALA II or African American for SAGE. Study exclusion criteria included the following: (1) any smoking within 1 year of the recruitment date; (2) ten or more pack-years of smoking; (3) pregnancy in the third trimester; and (4) a history of lung diseases other than asthma (for cases) or chronic illness (for cases and controls). Other Latino participants did not self-identify as Mexican American or Puerto Rican.

The local Institutional Review Board from the University of California, San Francisco Human Research Protection Program approved the studies (Institutional Review Board numbers 10-02877 (SAGE) and 10-00889 (GALA II)). All participants and their legal guardians provided written informed consent.

### WGS data and processing

Genomic DNA samples extracted from whole blood were sequenced as part of the Trans-Omics for Precision Medicine (TOPMed) WGS program^59^ and the Centers for Common Disease Genomics Genome Sequencing Program (GSP). WGS was performed at the New York Genome Center (NYGC) and Northwest Genomics Center (NWGC) on a HiSeq X system (Illumina) using a paired-end read length of 150 base pairs, with a minimum of 30× mean genome coverage. DNA sample handling, quality control, library construction, clustering, and sequencing, read processing and sequence data quality control have previously been described in detail^59^. All samples were jointly genotyped at the TOPMed Informatics Research Center. Variant calls were obtained from TOPMed data Freeze 8 VCF files generated based on the GRCh38 assembly. Variants with a minimum read depth of 10 (DP10) were used for analysis unless otherwise stated.

### RNA-seq data generation and processing

Total RNA was isolated from a PAXgene tube using a MagMAX for Stabilized Blood Tubes RNA Isolation Kit (4452306; Applied Biosystems). Globin depletion was performed using GLOBINcleasr Human (AM1980; Thermo Fisher Scientific). RNA integrity and yield were assessed using an Agilent 2100 Bioanalyzer (Agilent Technologies).

Total RNA was quantified using the Quant-iT RiboGreen RNA Assay Kit and normalized to 5 ng µl^−1^. An aliquot of 300 ng for each sample was transferred into library preparation, which was an automated variant of the Illumina TruSeq Stranded mRNA Sample Preparation Kit. This method preserves strand orientation of the RNA transcript. It uses oligo dT beads to select messenger RNA from the total RNA sample. It is followed by heat fragmentation and complementary DNA synthesis from the RNA template. The resultant complementary DNA then goes through library preparation (end repair, base A addition, adapter ligation and enrichment) using Broad-designed indexed adapters substituted in for multiplexing. After enrichment, the libraries were quantified with quantitative PCR using the KAPA Library Quantification Kit for Illumina Sequencing Platforms and then pooled equimolarly. The entire process was in 96-well format and all pipetting was done using either an Agilent Bravo or a Hamilton Starlet instrument.

Pooled libraries were normalized to 2 nM and denatured using 0.1 N NaOH before sequencing. Flow cell cluster amplification and sequencing were performed according to the manufacturer’s protocols using the HiSeq 4000. Each run was a 101-base pair paired-end read with an eight-base index barcode. Each sample was targeted to 50 million reads. Data were analyzed using the Broad Picard Pipeline, which includes demultiplexing and data aggregation.

RNA-seq reads were further processed using the TOPMed RNA-seq pipeline for year 3 and phase 5 RNA-seq data. Count-level data were generated using the GRCh38 human reference genome and GENCODE 30 for transcript annotation. Count-level quality control and normalization were performed following the GTEx project v8 protocol (https://gtexportal.org/home/methods). Sample-level quality control included the removal of RNA samples with an RNA integrity number of <6, genetically related samples (equally or more related than third-degree relative) and sex-discordant samples based on reported sex and *XIST* and *RPS4Y1* gene expression profiles. Count distribution outliers were detected as follows: (1) raw counts were normalized using the trimmed mean of *M* values method in edgeR^60^, as described in GTEx v8 protocol; (2) the log_2_-transformed normalized counts at the 25th percentile of every sample were identified (count_q25_); (3) the 25th percentile (Q25) of count_q25_ was calculated; and (4) samples were removed if their count_q25_ was lower than −4, as defined by visual inspection.

To account for hidden confounding factors, such as batch effects, technical and biological variation in the sample preparation and sequencing and/or data processing procedures, latent factors were estimated using the probabilistic estimation of expression residuals (PEER) method^61^. Optimization was performed according to an approach adopted by GTEx with the goal to maximize eQTL discovery^62^. A total of 50 (for AA, PR, MX and pooled samples) and 60 (for AFR_high_, AFR_low_, IAM_high_ and IAM_low_) PEER factors were selected for downstream analyses (Supplementary Fig. 6).

### Estimation of global and local genetic ancestry

Genetic principal components, global and local ancestry and kinship estimation on genetic relatedness were computed using biallelic SNPs with a PASS flag from TOPMed Freeze 8 DP10 data, as described previously^63,64^. Briefly, genotype data from European, African and Indigenous American ancestral populations were used as the reference panels for global and local ancestry estimation, assuming three ancestral populations.

Reference genotypes for European (HapMap CEU) and African (HapMap YRI) ancestries were obtained from the Axiom Genotype Data Set (https://www.thermofisher.com/us/en/home/life-science/microarray-analysis/microarray-data-analysis/microarray-analysis-sample-data/axiom-genotype-data-set). The CEU populations were recruited from Utah residents with Northern and Western European ancestry from the CEPH collection. The YRI populations were recruited from Yoruba in Ibadan, Nigeria. The Axiom Genome-Wide LAT 1 array was used to generate the Indigenous American ancestry reference genotypes from 71 Indigenous Americans (14 Zapotec, two Mixe and 11 Mixtec from Oaxaca and 44 Nahua from Central Mexico)^65,66^. ADMIXTURE was used with the reference genotypes in a supervised analysis assuming three ancestral populations. Global ancestry was estimated by ADMIXTURE^67^ in supervised analysis mode, whereas local ancestry was estimated by RFMIX version 2 with default settings^68^. Throughout this study, the local ancestry of a gene was defined as the number of ancestral alleles (zero, one or two) at the transcription start site.

Comparative analyses were performed based on two different sample grouping strategies, by self-identified race/ethnicity or by global ancestry. Self-identified race/ethnicity included four groups: AA, PR, MX and the pooling of AA, PR, MX and other Latino Americans (pooled). For groups defined by global ancestry, samples were grouped into high (>50%; AFR_high_ and IAM_high_) or low (<10%; AFR_low_ and IAM_low_) global African or Indigenous American ancestry. The sample size for each group is shown in Supplementary Table 1.

### *Cis*-heritability of gene expression

The genetic region of *cis*-gene regulation was defined by a 1-megabase (Mb) region flanking each side of the transcription start site (*cis*-region). *Cis*-heritability (*h*^2^) of gene expression was estimated using unconstrained genome-based restricted maximum likelihood (GREML)^69^ analysis (--reml-no-constrain) and estimation was restricted to common autosomal variants (MAF ≥ 0.01). Inverse-normalized gene expression was regressed on PEER factors and the residuals were used as the phenotype for GREML analysis. Sex and asthma case–control status were used as categorical covariates, while age at blood draw and the first five genetic principal components were used as quantitative covariates. *Cis*-heritability was estimated separately for each self-identified race/ethnicity group (AA, PR, MX and pooled) and groupings based on global (AFR_high_, AFR_low_, IAM_high_ and IAM_low_) and local ancestry (described below). Differences in the distribution of *h*^2^ and genetic variance (*V*_G_) between groups were tested using two-sided Wilcoxon tests. Parallel analyses were also conducted for Indigenous American ancestry (IAM/IAM versus EUR/EUR and IAM/IAM versus IAM/EUR).

The following sensitivity analyses were conducted using GCTA: (1) using the same sample size in each self-identified group (*n* = 600); and (2) partitioning heritability and genetic variance by two MAF bins (0.01–0.10 and 0.1–0.5). We also estimated heritability using the LDAK-Thin model^70^, following the recommended genetic relatedness matrix processing. Thinning of duplicate SNPs was performed using the arguments --window-prune .98 --window-kb 100. The direct method was applied to calculate kinship using the thinned data and, lastly, generalized restricted maximum likelihood (REML) was used to estimate heritability.

### Association of global and local ancestry with gene expression

Methods from Gay et al.^58^ were modified to identify genes associated with global and local ancestry. In step 1, inversed normalized gene expression was regressed on age, sex and asthma status (model 0). In step 2, the residuals from model 0 were regressed on global ancestry (model 1). In step 3, the residuals from model 1 were regressed on local ancestry (model 2) to identify genes that are associated with local ancestry. An FDR of 0.05 was applied to steps 2 and 3 separately to identify genes that were significantly associated with global and/or local ancestry. Steps 1–3 were run separately for African and Indigenous American ancestry. For heritable genes that were associated with global and/or local ancestry, a joint model of regressing global and local ancestry from residuals from model 0 was also examined to assess the percentage of variance of gene expression explained by global and/or local ancestry.

### Identification of eGenes, *cis*-eQTLs and ancestry-specific *cis*-eQTLs

FastQTL^71^ was used to process raw gene counts and identify eQTLs, according to the GTEx v8 pipeline (https://github.com/broadinstitute/gtex-pipeline). Age, sex, asthma status, the first five genetic ancestry principal components and PEER factors were used as covariates for FastQTL analysis. To account for multiple testing across all tested genes, Benjamini–Hochberg correction was applied to the beta-approximated *P* values from the permutation step of FastQTL. For each gene with a significant beta-approximated *P* value at an FDR of <0.05, a nominal *P* value threshold was estimated using the beta-approximated *P* value. *Cis*-eQTLs were defined as genetic variants with nominal *P* values less than the nominal *P* value threshold of the corresponding gene. eGenes were defined as genes with at least one eQTL. To summarize the number of independent *cis*-eQTLs in each ancestry group, linkage disequilibrium clumping was performed using PLINK (--clump-kb 1000 --clump-r2 0.1) using gene-specific *P* value thresholds.

*Trans*-eQTLs were identified using the same protocol as in GTEx v8 (ref. ^2^). *Trans*-eQTLs were defined as eQTLs that were not located on the same chromosome as the gene. Only protein-coding and long intergenic noncoding RNA genes and SNPs on autosomes were included in the analyses. Briefly, linear regression on the expression of the gene was performed in PLINK2 (version v2.00a3LM; released 28 March 2020) using SNPs with MAF ≥ 0.05 and the same covariates as in *cis*-eQTL discovery. Gene and variant mappability data (GRCh38 and GENCODE v26) were downloaded from Saha and Battle^72^ for the following filtering steps: (1) keep gene–variant pairs that pass a *P* value threshold of 1 × 10^-5^; (2) keep genes with a mappability of ≥0.8; (3) remove SNPs with a mappability of <1; and (4) remove a *trans*-eQTL candidate if genes within 1 Mb of the SNP candidate cross-map with the *trans*-eGene candidate. The Benjamini–Hochberg procedure was applied to control for the FDR at the 0.05 level using the smallest *P* value (multiplied by 10^−6^) from each gene. An additional filtering step was applied for the AFR_high_ and IAM_high_ groups. For AFR_high_, all *trans*-eQTLs detected in AFR_low_ group were removed and the resulting *trans*-eQTLs were referred to as filtered AFR_high_ *trans*-eQTLs. Similarly, for the IAM_high_ group, all *trans*-eQTLs detected in the IAM_low_ group were removed and the resulting *trans*-eQTLs were referred to as filtered IAM_high_ *trans*-eQTLs. Filtered AFR_high_ *trans*-eQTLs were checked for the presence of filtered IAM_high_ *trans*-eQTLs and vice versa. Linkage disequilibrium clumping was performed using PLINK (v1.90b6.26 --clump-kb 1000 --clump-r2 0.1 --clump-p1 0.00000005 --clump-p2 1) to group *trans*-eQTLs into independent signals.

Mapping of anc-eQTLs was performed in participants stratified by high and low global African and Indigenous American ancestry. We developed a framework to identify anc-eQTLs by focusing on the lead eQTL signal for each gene and comparing fine-mapped 95% credible sets between high (>50%) and low (<10%) global ancestry groups (AFR_high_ versus AFR_low_ and IAM_high_ versus IAM_low_). Sensitivity analyses were conducted using >70% as the cut-off for the AFR_high_ and IAM_high_ groups. Anc-eQTLs were classified into three tiers as described below, based on population differences in allele frequency, linkage disequilibrium and effect size (Fig. 3a). For every protein-coding and heritable eGene (GCTA *h*^2^ likelihood ratio test *P* value < 0.05), the lead eQTL signal was identified using CAVIAR^73^, assuming one causal locus (*c* = 1). The 95% credible sets of eQTLs in the high and low global ancestry groups were compared to determine whether there was any overlap. Variants from nonoverlapping 95% credible sets were further classified as tier 1 anc-eQTLs based on allele frequency differences, or tier 2 after additional fine-mapping using PESCA^22^. For genes with overlapping 95% credible sets, tier 3 anc-eQTLs were detected based on effect size heterogeneity.

eQTLs identified in the AFR_high_ or IAM_high_ groups that were common (MAF ≥ 0.01) in the high groups but rare (MAF < 0.01) or monomorphic in the AFR_low_ or IAM_low_ groups were classified as tier 1. If the eQTLs were detected at an MAF of ≥0.01 in both the high and low ancestry groups, they were further fine-mapped using PESCA^22^, which tests for differential effect sizes while accounting for linkage disequilibrium between eQTLs. Preprocessing for the PESCA analyses involved linkage disequilibrium pruning at *r*^2^ > 0.95. All eQTL pairs with *r*^2^ > 0.95 were identified in both the high and low groups and only those pairs common to both groups were removed. For each eQTL, PESCA estimated three posterior probabilities: specific to the AFR_high_ or IAM_high_ group (PP_high_); specific to the AFR_low_ or IAM_low_ group (PP_low_); or shared between the two groups (PP_shared_). Tier 2 anc-eQTLs were selected based on the following criteria: (1) all variants in the credible set have (PP_high_ > PP_low_) and (PP_high_ > PP_shared_); and (2) PP_high_ > 0.8. The tier 3 class was based on evidence of significant heterogeneity in eQTL effect size, defined as a Cochran’s *Q* *P* value of <0.05/*n*_gene_, where *n*_gene_ was the number of genes tested. Since we assume that the 95% credible set corresponds to a single lead eQTL signal, all eQTLs in the credible set were required to have a significant heterogeneous effect size to be classified as tier 3 anc-eQTLs.

To systematically assess the overlap in eQTL signals identified in our study and trait-associated loci, we colocalized eQTL summary statistics with GWAS results from PAGE. Colocalization was performed using COLOC^74^ within a linkage disequilibrium window of 2 Mb centered on the eQTL with the lowest GWAS *P* value. For each eQTL–trait pair, a posterior probably of a shared causal signal (PP_4_) of >0.80 was interpreted as strong evidence of colocalization.

### Development and application of multi-ancestry TWAS models

Gene prediction models for *cis*-gene expression were generated using common variants and elastic net modeling implemented in the PredictDB v7 pipeline (https://github.com/hakyimlab/PredictDB_Pipeline_GTEx_v7). Models were filtered by nested cross-validation prediction performance and heritability *P* value (rho_avg > 0.1, zscore_pval < 0.05 and GCTA *h*^2^ *P* value < 0.05). Sensitivity analyses were performed by generating gene prediction models that included the number of ancestral alleles as covariates to account for local ancestry in the *cis*-region. In the AA group, one covariate indicating the count of African ancestral alleles was used, whereas in the PR, MX and pooled groups, two additional covariates indicating the numbers of European and Indigenous American ancestral alleles were used.

Out-of-sample validation of the gene expression prediction models was done using 598 individuals from the African American asthma cohort SAPPHIRE^31^. Predicted gene expression from SAPPHIRE genotypes was generated using the predict function from MetaXcan. Genotypes of SAPPHIRE samples were generated by whole-genome sequencing through the TOPMed program and were processed in the same way as for GALA II and SAGE. RNA-seq data from SAPPHIRE were generated as previously described^75^ and were normalized using the trimmed mean of *M* values method in edgeR. Predicted and normalized gene expression data were compared to generate correlation *r*^2^ values.

To assess the performance of the resulting GALA II/SAGE models, we conducted TWASs of 28 traits using GWAS summary statistics from the PAGE Consortium study by Wojcik et al.^21^. Analyses were performed using S-PrediXcan with whole-blood gene prediction models from GALA II and SAGE (GALA II/SAGE models) and GTEx v8, as well as monocyte gene expression models from MESA^3^. In the UKB, we conducted TWASs of 22 blood-based biomarkers and quantitative traits using GALA II/SAGE models generated in African Americans (GALA II/SAGE AA) and GTEx v8 whole blood. Each set of TWAS models was applied to publicly available GWAS summary statistics (Pan-UKB team; https://pan.ukbb.broadinstitute.org) from participants of predominantly European ancestry (UKB EUR) and African ancestry (UKB AFR). Ancestry assignment in UKB was based on a random forest classifier trained on the merged 1000 Genomes and Human Genome Diversity Project reference populations. The classifier was applied to UKB participants projected into the 1000 Genomes and Human Genome Diversity Project principal components.

### Reporting summary

Further information on research design is available in the Nature Portfolio Reporting Summary linked to this article.

## Online content

Any methods, additional references, Nature Portfolio reporting summaries, source data, extended data, supplementary information, acknowledgements, peer review information; details of author contributions and competing interests; and statements of data and code availability are available at 10.1038/s41588-023-01377-z.

## Supplementary information

Supplementary Information Supplementary Figs. 1–7 and Tables 1–10 and 13–15.

Reporting Summary

Supplementary Tables 11 and 12 Table 11: Ancestry-specific eQTLs that were reported in the NHGRI-EBI GWAS catalog. Table 12: Colocalization results for identified ancestry-specific eQTLs and 28 traits in PAGE. Trait–eQTL pairs with a PP of colocalization (PP.H4.abf) of >0.50 are reported.

## Data Availability

WGS and RNA-seq data for the GALA II, SAGE and SAPPHIRE studies, generated as part of the NHLBI TOPMed program, are available from the Database of Genotypes and Phenotypes under accession numbers phs000920 (GALA II), phs000921 (SAGE) and phs001467 (SAPPHIRE). Comprehensive phenotypic data for GALA II study participants are available through the Database of Genotypes and Phenotypes (phs001180). Summary statistics for *cis*- and *trans*-eQTLs, a catalog of ancestry-specific eQTLs, TWAS models developed using data from GALA II and SAGE participants, and normalized individual-level gene expression data have been posted in a public repository at 10.5281/zenodo.7735723.

## Code availability

A description of the RNA-seq harmonization pipeline is available at 10.5281/zenodo.7735723. Scripts for performing heritability analyses, eQTL mapping and TWAS model development are available at https://github.com/angelcymak/gala2_sage_eQTL. Heritability analyses were performed using GCTA (https://yanglab.westlake.edu.cn/software/gcta/#GREMLanalysis). FastQTL analysis was conducted following the GTEx v8 pipeline (https://github.com/broadinstitute/gtex-pipeline). TWAS models were generated following the PredictDB v7 pipeline (https://github.com/hakyimlab/PredictDB_Pipeline_GTEx_v7).
