# Supplementary material for: Gene expression in African Americans, Puerto Ricans and Mexican Americans reveals ancestry-specific patterns of genetic architecture
Source: Nat Genet. 2023 May 25;55(6):952–63. doi: 10.1038/s41588-023-01377-z (PMC10260401; doi:10.1038/s41588-023-01377-z)
Supplement: Supplementary file 1 — Supplementary Figs. 1–7 and Tables 1–10 and 13–15. [file 41588_2023_1377_MOESM1_ESM.pdf]

nature genetics

Article https://doi.org/10.1038/s41588-023-01377-z

Gene expression in African Americans, Puerto Ricans and Mexican Americans reveals ancestry-specific patterns of genetic architecture

In the format provided by the authors and unedited

Supplementary Figure 1: Distribution of global genetic ancestry in within self-identified race/ethnicity groups. Each panel visualizes the number of individuals within deciles of global genetic ancestry for European (EUR), African (AFR), and Indigenous American (IAM) ancestry. Stacked bar plots show the number of individuals from self-reported race/ethnicity groups within each ancestry decile. Vertical lines show the thresholds for deriving ancestry-based groups for eQTL comparisons. Figure created with BioRender.com

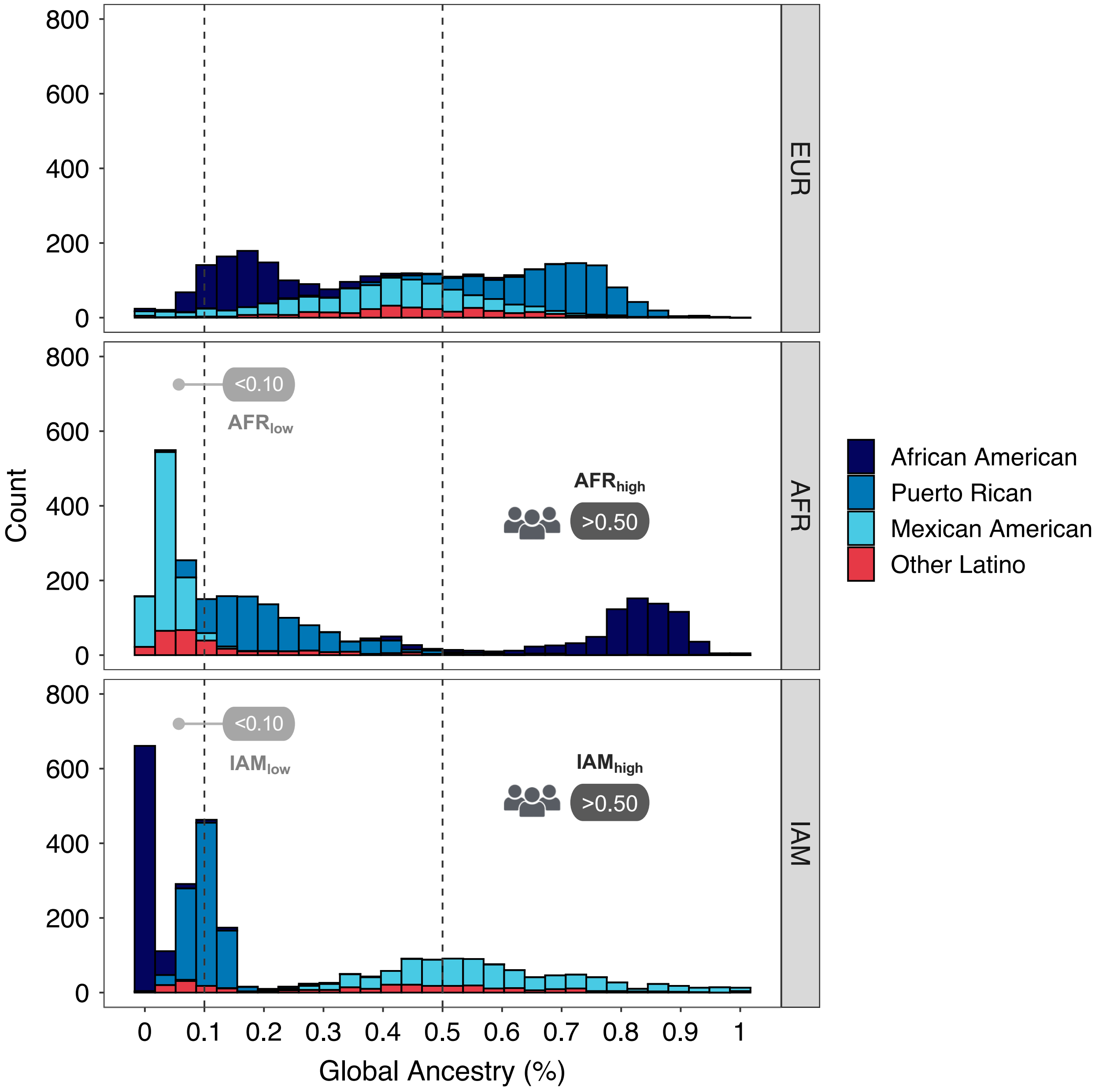

Supplementary Figure 2: Association of global and local ancestry with gene expression levels. Stepwise local regression was used to identify genes for which global and/or local ancestry had a significant (FDR<0.05) effect on transcript levels., the variance in transcript levels accounted for by **a,** African and **b,** Indigenous American ancestry. In each panel, inset plots visualize the 0-15% range on the y-axis, without outliers while the full range percentage variance explained are shown in the top panel. Red box highlights the zoomed region as shown in the bottom panel. Analyses are stratified by self-reported race/ethnicity: African American (AA), Puerto Rican (PR), and Mexican American (MX).

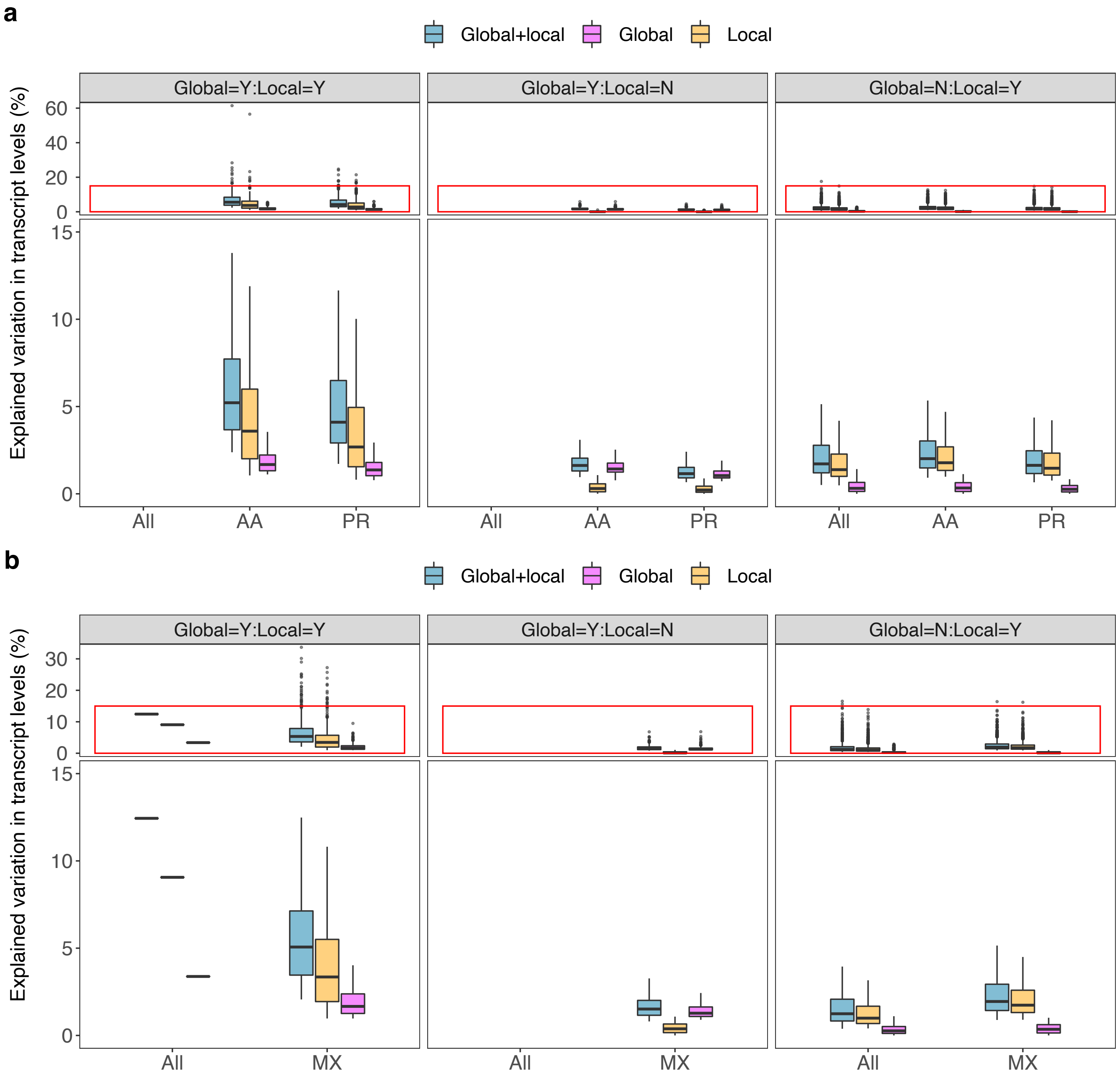

Supplementary Figure 3: Overlap of eGenes between self-identified race/ethnicity groups. Venn diagrams show the overlap in genes with at least one statistically significant eQTL (eGenes) between GALA/SAGE participants. Overlap was tabulated for all genes and for protein-coding genes only.

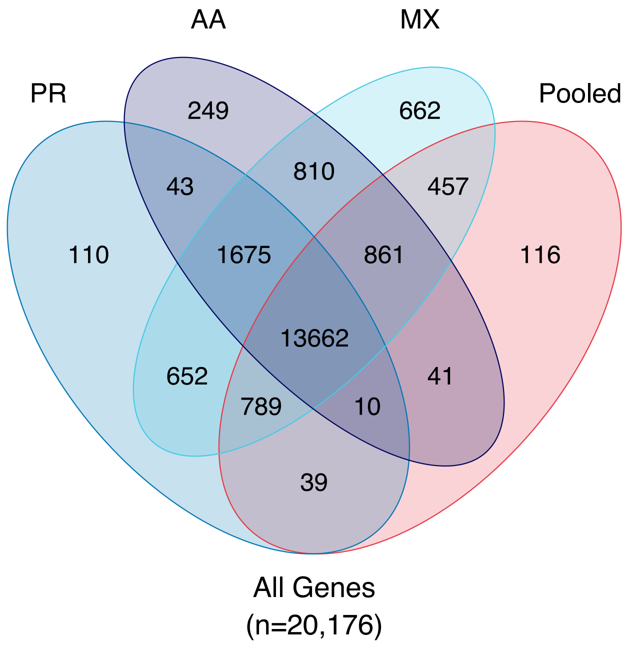

Supplementary Figure 4: Cross validation R^2 of transcriptome prediction models. Cross validation (CV) R^2 for each gene is overlaid on the *cis*-heritability (h^2), indicated by the black point, with 95% confidence intervals as gray lines. Elastic net transcriptome prediction models were generated using the PredictDB analytic pipeline. Genes are sorted by increasing h^2 values. **a-d,** Prediction model performance is shown for models trained in African American, Puerto Rican, Mexican American participants, and the pooled sample that also included individuals self-identified as Other Latino.

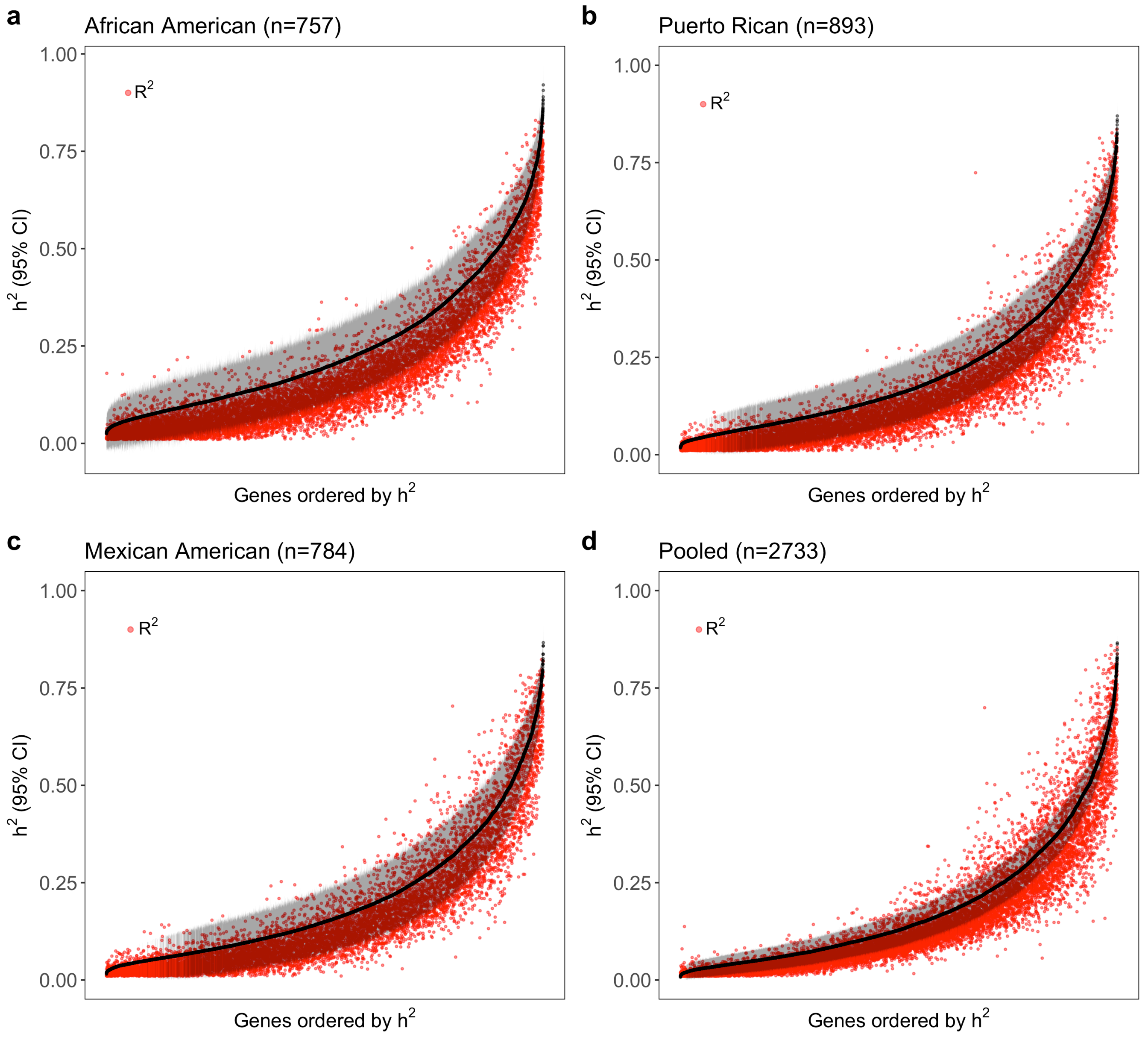

Supplementary Figure 5: Out of sample validation of TWAS models in the SAPPHIRE. a, Admixture plots for the SAPPHIRE validation study and each of the training samples used to develop the TWAS models. **b,** Correlations between predicted and observed gene expression levels are shown for 5254 genes with prediction models available in GTEx and GALA/SAGE. For each set of TWAS models, violin plots visualize the distribution of correlation coefficients, with circles indicating the median correlation, and crossbars showing the interquartile range (IQR) from the 25th to the 75th percentile. Two-sided p-values are based on the Wilcoxon test.

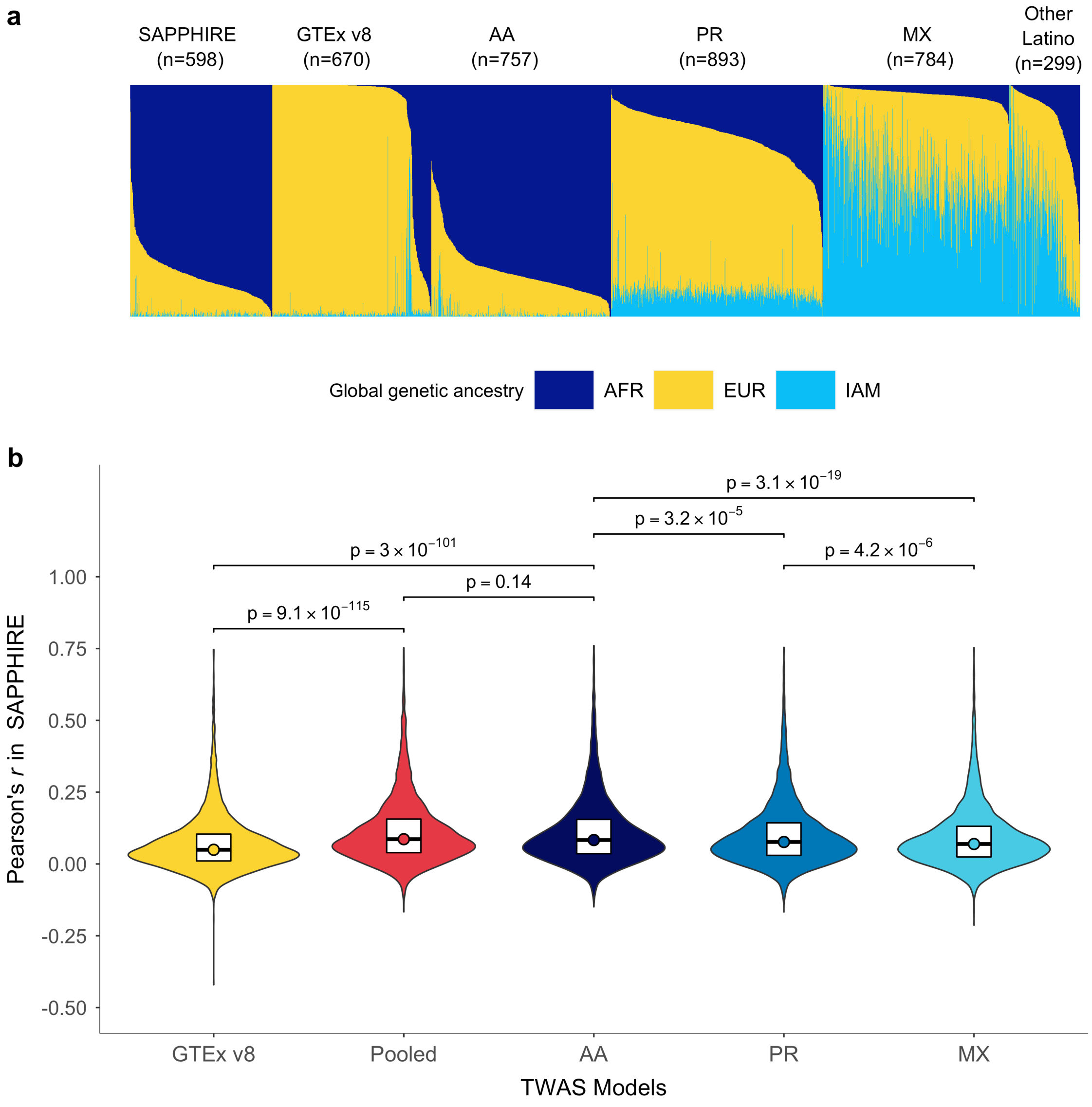

		GTEx v8	Pooled	AA	PR	MX
Intersection of genes		5254	5254	5254	5254	5254
Validation: Pearson's *r* >0	nGene	4224	4739	4713	4646	4544
	(%)	(80.4)	(90.2)	(0.897)	(88.4)	(86.5)
Pearson's *r*	Mean	0.070	0.113	0.111	0.102	0.092
	Median	0.049	0.086	0.083	0.077	0.069
	IQR	0.010 - 0.104	0.039 - 0.156	0.036 - 0.155	0.030 - 0.143	0.025 - 0.131

Supplementary Figure 6: Conditional TWAS of neutrophil counts in UK Biobank. Given the extensive LD in the region around the *ACKR1* (atypical chemokine receptor) gene, that is the basis of the Duffy blood group system, we compared results when applying GALA/SAGE African American TWAS models to marginal GWAS results and GWAS conditioned on rs2814778, the Duffy null variant responsible for constitutively lower neutrophil levels. GWAS results for neutrophil counts are based on 6115 African ancestry subjects from the UK Biobank. Genes with FDR<0.05 are highlighted in orange. The dotted line corresponds to the Bonferroni adjusted p-value threshold of 4.5×10^{-5} = 0.05/1120 genes tested. Both FDR and Bonferroni only account for local multiple comparisons within chromosome 1.

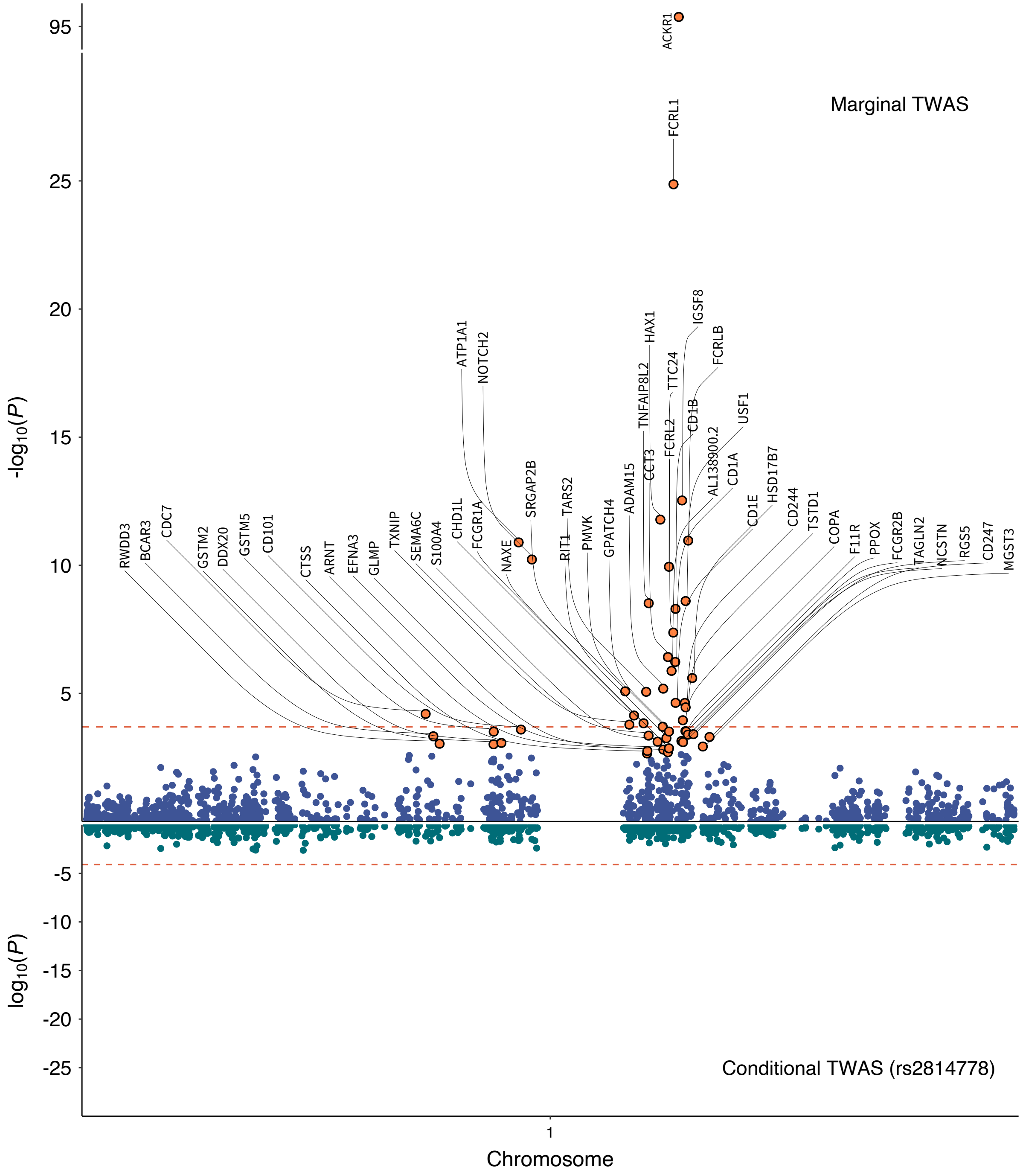

Supplementary Figure 7: Selection of PEER factors for eQTL analysis. The number of **a,** eQTLs and **b,** eGenes detected as a function of PEER factors included as covariates in analyses stratified by self-reported race/ethnicity groups: African Americans (AA), Puerto Ricans (PR), and Mexican Americans (MX). The number of **c,** eQTLs and **d,** eGenes detected as a function of PEER factors included as covariates in analyses stratified genetic ancestry groups: >50% African ancestry (AFR_{high}), <10% African ancestry (AFR_{low}), >50% Indigenous American ancestry (IAM_{high}), <10% Indigenous American ancestry (IAM_{low}). Vertical dashed lines indicate the number of PEER factors selected for the final analysis with the goal of maximizing eQTL discovery.

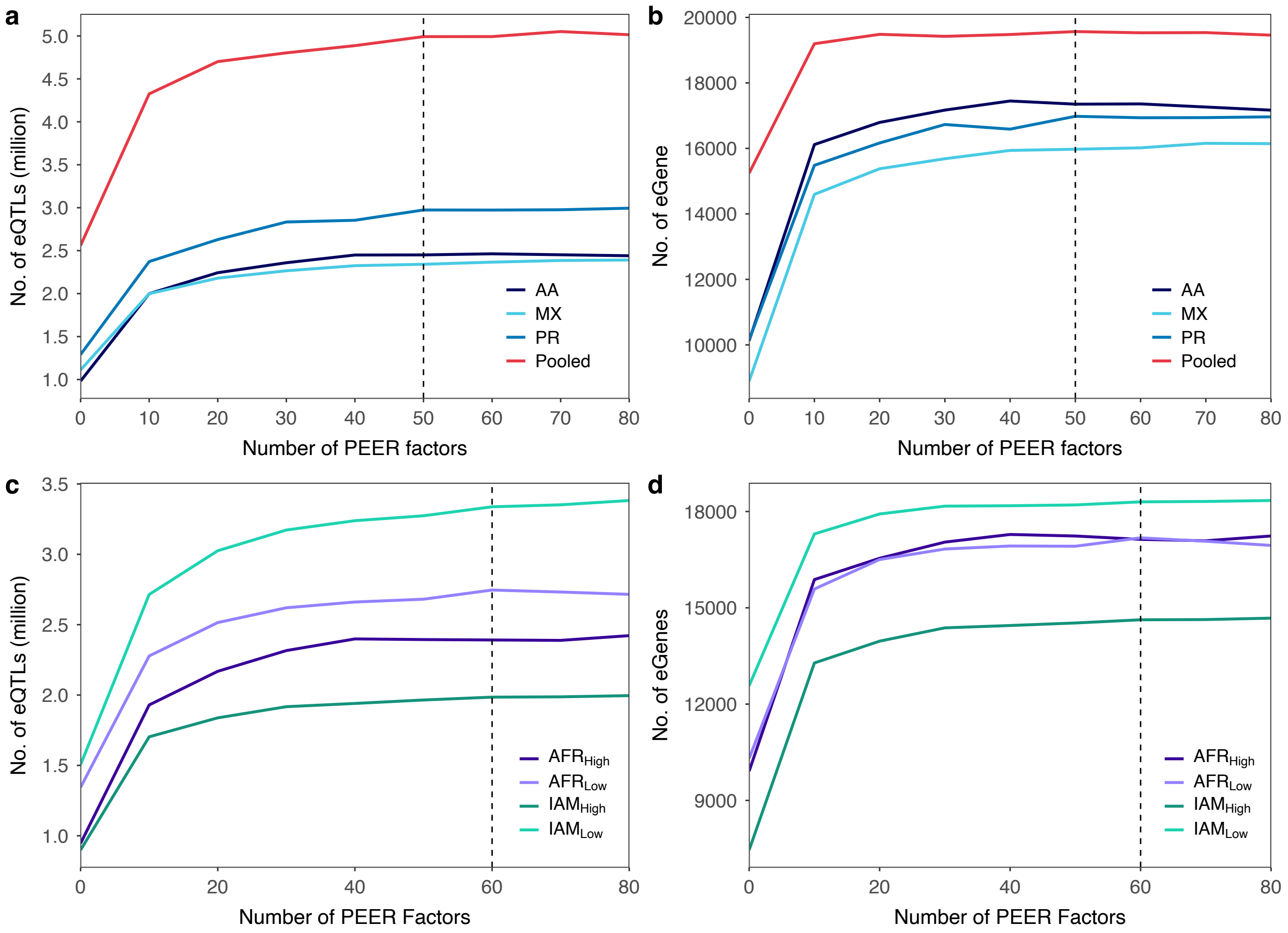

Supplementary Table 1: Study population. The total number of individuals with whole genome and RNA sequencing data from the Genes-environments and Admixture in Latino Americans (GALA II) and the Study of African Americans, Asthma, Genes, and Environments (SAGE) included in the present analysis. Participant groups based on race/ethnicity are mutually exclusive. Groupings based on genetic ancestry are non-overlapping only within African (AFR) or Indigenous American (IAM) strata. For example, a subject with 85% AFR ancestry will be included in the AFR_{high} analysis and may also be included in the IAM_{low} analysis, if they meet the <10% IAM ancestry criterion.

	Participant Groups	Sample Size
Self-identified race/ ethnicity	African American	757
	Puerto Rican	893
	Mexican American	784
	Other Latino	299
	Total/Pooled	2733
Global genetic ancestry	AFR_{high} (AFR > 50%)	721
	AFR_{low} (AFR < 10%)	1011
	AFR sub-total	1732
	IAM_{high} (IAM > 50%)	610
	IAM_{low} (IAM < 10%)	1257
	IAM sub-total	1867

Supplementary Table 2: *Cis*-heritability (h^2) and genetic variance (V_G) of gene expression stratified by self-identified race/ethnicity. GCTA analyses were restricted to common variants, with MAF ≥0.01 in each population, within 1MB flanking regions of the transcription start site. Estimates of h^2 and V_G are summarized across the intersection of genes (nGene) with GCTA results available in all populations. All p-values are two-sided.

	African American (n=757)	Puerto Rican (n=893)	Mexican American (n=784)	Pooled (n=2733)
nGene	17,657	17,657	17,657	17,657
h^2				
Mean	0.170	0.142	0.130	0.148
Median	0.111	0.080	0.066	0.087
IQR	0.039 – 0.252	0.026 – 0.204	0.019 – 0.184	0.030 – 0.211
V_G				
Mean	0.059	0.052	0.044	0.054
Median	0.025	0.020	0.014	0.020
IQR	0.006 – 0.073	0.005 – 0.060	0.003 – 0.047	0.006 – 0.062
Mean global ancestry proportion				
AFR	0.80	0.22	0.04	0.32
IAM	0.01	0.10	0.57	0.24
Wilcoxon p-value of h^2 comparison between groups				
AA	-	1.7×10^{-69}	1.8×10^{-160}	1.9×10^{-34}
PR	-	-	3.1×10^{-24}	2.1×10^{-10}
MX	-	-	-	1.8×10^{-64}
Wilcoxon p-value of V_G comparison between groups				
AA	-	4.3×10^{-23}	2.3×10^{-148}	-
PR	-	-	2.0×10^{-62}	-
MX	-	-	-	-

Abbreviations

AFR	African ancestry
IAM	Indigenous American ancestry
IQR	Interquartile range extending from the 25th to 75th percentiles
Pooled	Analysis includes AA, PR, MX, and other Latinos

Supplementary Table 3: ***Cis*-heritability (h^2) and genetic variance (V_G) of gene expression stratified by global genetic ancestry.** GCTA analyses were restricted to common variants, with MAF ≥0.01 in each population, within 1MB flanking regions of the transcription start site. Individuals were stratified based on proportion. Individuals with >50% global genetic African ancestry (AFR_{high}) were compared to those with <10% (AFR_{low}). Individuals with >50% global genetic Indigenous American ancestry (IAM_{high}) were compared to those with <10% (IAM_{low}). Estimates of h^2 and V^G are summarized across the intersection of genes (nGene) with GCTA results available in all genetic ancestry groups. All p-values are two-sided.

	**AFR_{high} (n=721)**	**AFR_{low} (n=1011)**	**IAM_{high} (n=610)**	**IAM_{low} (n=1257)**
nGene	18,725	18,725	18,725	18,725
h^2				
Mean	0.167	0.129	0.123	0.152
Median	0.107	0.065	0.062	0.091
IQR	0.037 – 0.247	0.019 – 0.182	0.016 – 0.176	0.031 – 0.221
V_G				
Mean	0.058	0.046	0.041	0.055
Median	0.024	0.014	0.013	0.022
IQR	0.006 – 0.072	0.003 – 0.048	0.002 – 0.045	0.006 – 0.066
Mean global ancestry proportion				
AFR	0.82	0.04	0.04	0.58
IAM	0.01	0.54	0.67	0.04
Wilcoxon p-value of h^2 comparison between groups				
AFR_{high}	-	4.2×10^{-140}	-	-
IAM_{high}	-	-	-	5.8×10^{-120}
Wilcoxon p-value of V_G comparison between groups				
AFR_{high}	-	8.0×10^{-114}	-	-
IAM_{high}	-	-	-	8.3×10^{-173}

Abbreviations

AFR	African ancestry
IAM	Indigenous American ancestry
IQR	Interquartile range extending from the 25th to 75th percentiles
Pooled	Analysis also includes participants self-identified as Other Latino

Supplementary Table 4: Comparison of V_G stratified by local genetic ancestry. GCTA analyses were restricted to common variants, with MAF ≥0.01 in each population, within 1MB flanking regions of the transcription start site. For each gene, individuals were classified into local ancestry groups, L1 and L2, based on the ancestry at the transcription start site. The number of genes (nGene) for which GCTA models successfully converged and produced reliable heritability (h^2) and genetic variance (V_G) estimates is reported for each analysis. Genes were not filtered based on statistically significant heritability. Wilcoxon p-values comparing h^2 and V_G between L1 and L2 are two-sided.

Population	Local ancestry group				Mean h^2		P-value	Mean V_G		P-value
	N	nGene	L1	L2	L1	L2		L1	L2	
Pooled	516	17,866	AFR/AFR	AFR/EUR	0.153	0.142	1.4×10^{-14}	0.053	0.049	2.0×10^{-7}
African American	202	19,224	AFR/AFR	AFR/EUR	0.143	0.137	6.2×10^{-5}	0.041	0.039	5.7×10^{-5}
Puerto Rican	242	18,570	AFR/EUR	EUR/EUR	0.129	0.108	1.8×10^{-32}	0.041	0.033	1.4×10^{-28}
Pooled	359	10,566	IAM/IAM	IAM/EUR	0.108	0.119	1.6×10^{-7}	0.033	0.038	1.6×10^{-8}
Mexican American	262	18,194	IAM/IAM	IAM/EUR	0.101	0.117	3.4×10^{-14}	0.029	0.035	7.7×10^{-11}

Abbreviations

AFR	African ancestry
IAM	Indigenous American ancestry
Pooled	Analysis also includes participants self-identified as Other Latino

Supplementary Table 5: *Cis*-heritability (h^2) estimated using LDAK-Thin. Analyses were restricted to common variants, with MAF ≥0.01 in each population, within 1MB flanking regions of the transcription start site. Individuals were stratified based on proportion. Individuals with >50% global genetic African ancestry (AFR_{high}) were compared to those with <10% (AFR_{low}). Individuals with >50% global genetic Indigenous American ancestry (IAM_{high}) were compared to those with <10% (IAM_{low}). Estimates of h^2 are summarized across the intersection of genes (nGene) with LDAK results available in all genetic ancestry groups. All p-values are two-sided.

	**African American (n=757)**	**Puerto Rican (n=893)**	**Mexican American (n=784)**	**Pooled (n=2733)**
nGene	18,261	18,261	18,261	18,261
h^2				
Mean	0.157	0.136	0.125	0.146
Median	0.094	0.071	0.059	0.081
IQR	0.029 – 0.234	0.020 – 0.194	0.016 – 0.176	0.024 – 0.213
Wilcoxon p-value of h^2 comparison between groups				
AA	-	2.6×10^{-43}	1.8×10^{-104}	3.0×10^{-9}
PR	-	-	1.9×10^{-16}	2.7×10^{-17}
MX	-	-	-	6.1×10^{-65}
	**AFR_{high} (n=721)**	**AFR_{low} (n=1011)**	**IAM_{high} (n=610)**	**IAM_{low} (n=1257)**
nGene	18,475	18,475	18,475	18,475
h^2				
Mean	0.166	0.132	0.125	0.157
Median	0.104	0.066	0.062	0.093
IQR	0.035 – 0.246	0.020 – 0.187	0.017 – 0.179	0.032 – 0.229
Wilcoxon p-value of h^2 comparison between groups				
AFR_{high}	-	1.9×10^{-117}		
IAM_{high}	-	1.0×10^{-122}		

Abbreviations

AFR	African ancestry
IAM	Indigenous American ancestry
IQR	Interquartile range extending from the 25th to 75th percentiles
Pooled	Analysis also includes participants self-identified as Other Latino

Supplementary Table 6: Number of heritable genes significantly associated with global and local ancestry. Analyses were restricted to heritable and autosomal genes with local ancestry estimates, and

populations with sufficient variability for a given ancestry comparison. The number of association genes is tabulated for all combinations of global and local ancestry associations. For example, group $AFR_{G=Y.L=Y}$ (global ancestry=Y and local ancestry=Y) includes genes that are associated with both global and local African ancestry at FDR < 0.05 level.

**Ancestry Associations**		**FDR < 0.05**		**African American (n=757)**		**Puerto Rican (n=893)**		**Mexican American (n=784)**	
		**Global**	**Local**	**nGene**	**%**	**nGene**	**%**	**nGene**	**%**
AFR	$AFR_{G=Y:L=Y}$	Yes	Yes	204	1.5	334	2.5	-	-
	$AFR_{G=Y:L=N}$	Yes	No	326	2.4	589	4.5	-	-
	$AFR_{G=N:L=Y}$	No	Yes	1,201	8.9	1,443	10.9	-	-
	$AFR_{G=N:L=N}$	No	No	11,833	87.2	10,856	82.1	-	-
IAM	$IAM_{G=Y:L=Y}$	Yes	Yes	-	-	-	-	389	3.1
	$IAM_{G=Y:L=N}$	Yes	No	-	-	-	-	353	2.8
	$IAM_{G=N:L=Y}$	No	Yes	-	-	-	-	1,228	9.8
	$IAM_{G=N:L=N}$	No	No	-	-	-	-	10,559	84.3
No. of heritable autosomal genes				13,596	-	13,260	-	12,562	-
No. of genes analyzed				13,564	-	13,222	-	12,529	-

Abbreviations

AFR	African ancestry
IAM	Indigenous American ancestry
IQR	Interquartile range extending from the 25th to 75th percentiles
Pooled	Analysis also includes participants self-identified as Other Latino

Supplementary Table 7: eQTLs and eGenes identified from each population. Results of FastQTL analyses conducted in GALA II / SAGE participants grouped based on self-identified race/ethnicity and genetic ancestry. Individuals were stratified based on proportion. Individuals with >50% global genetic African ancestry (AFR_{high}) were compared to those with <10% (AFR_{low}). Individuals with >50% global genetic Indigenous American ancestry (IAM_{high}) were compared to those with <10% (IAM_{low}).

Populations	N	Number of eQTLs	Number of eQTL-gene pairs	Number of eGenes
Pooled (Total)	2733	4,984,220	13,402,207	19,567
African American	757	2,448,802	4,399,353	17,336
Puerto Rican	893	2,970,694	6,032,429	16,975
Mexican American	784	2,333,522	5,232,074	15,938
Genetic ancestry groups				
AFR_{high}	721	2,389,968	4,260,212	17,123
AFR_{low}	1011	2,736,501	6,601,500	17,146
IAM_{high}	610	1,979,263	4,180,137	14,579
IAM_{low}	1257	3,334,768	6,831,948	18,297
Genetic ancestry groups (equal sample size)				
AFR_{high}	600	1,975,039	3,339,661	16,110
AFR_{low}		1,888,196	3,880,554	14,344
IAM_{high}		1,953,964	4,104,553	14,419
IAM_{low}		1,707,612	2,841,161	14,866

Abbreviations

AFR	African ancestry
IAM	Indigenous American ancestry
Pooled	Analysis also includes participants self-identified as Other Latino

Supplementary Table 8: Gene pre-filtering for ancestry-specific eQTL analysis. Significant *cis*-heritability, statistical significance of heritability estimates was determined using LRT p-value provided by GCTA. A total of 9609 and 8515 genes were used as the input to the ancestry-specific eQTL classification pipeline.

	**AFR**	**IAM**
Input number of genes	20,135	20,135
Protein coding genes (autosomal)	13,535	13,535
Significant *cis*-heritability in high group (LRT $p < 0.05$)	10,225	8,889
eGene in high ancestry group (>50% AFR or IAM)	10,077	8,594
95% credible sets generated using CAVIAR in both high and low (<10% AFR or IAM) ancestry group	9,609	8,515

Abbreviations

AFR	African ancestry
IAM	Indigenous American ancestry

Supplementary Table 9: Classification of ancestry-specific eQTLs (anc-eQTLs) using 50% global ancestry cutoff. Analyses were restricted to heritable genes described in Supplementary Table 8. Comparisons were conducted using >50% as the cut-off for AFR_{high} and IAM_{high} groups. Tier 1 represents the most ancestry-specific eQTL class, followed by Tier 2 anc-eQTLs. Tier 3 eQTLs were detected within overlapping 95% credible sets that are shared between ancestry groups and represent the least ancestry-specific class.

	**AFR_{high} (n=721) vs. AFR_{low} (n=1011)**			**IAM_{high} (n=610) vs. IAM_{low} (n=1257)**		
	**nGene**	**%**	**Gene-eQTL pairs AFR_{high}**	**nGene**	**%**	**Gene-eQTL pairs IAM_{high}**
Genes analyzed	9609	100	3,020,690	8515	100	3,015,261
No overlap in 95% credible set[1,2]	4551	47.4	1,257,678	3160	37.1	938,278
Tier 1	2695	28.0	41,102	562	6.6	3,938
PESCA input	2921	30.4	41,632	2999	35.2	98,149
Tier 2	109	1.1	112	33	0.4	36
Overlapping 95% credible set[3]	5058	52.6	1,763,012	5355	62.9	2,076,983
Tier 3	196	2.0	894	88	1.0	420
**Union of Tiers 1-3**	**2961**	**30.8**	**42,108**	**679**	**8.0**	**4394**

[1] Tier 1 eQTLs includes variants that are rare (MAF<0.01) or monomorphic in the low ancestry (<10%) group

[2] Tier 2 eQTLs were identified using fine-mapping using PESCA and include variants with posterior probability (PP_{high})>0.80 of being specific to AFR_{high} or IAM_{high} and have PP_{high}>PP_{low}

[3] Tier 3 eQTLs show effect size heterogeneity based on Cochran's Q test (P_Q<0.05/number of genes tested)

Abbreviations

AFR	African ancestry
IAM	Indigenous American ancestry

Supplementary Table 10: Classification of ancestry-specific eQTLs (anc-eQTLs) using 70% global ancestry as cutoff. Analyses were restricted to heritable genes described in Supplementary Table 8. Comparisons were conducted using >70% as the cut-off for AFR_{high} and IAM_{high} groups. Tier 1 represents the most ancestry-specific eQTL class, followed by Tier 2 anc-eQTLs. Tier 3 eQTLs were detected within overlapping 95% credible sets that are shared between ancestry groups and represent the least ancestry-specific class.

	AFR_{high} (n=653) vs. AFR_{low} (n=1011)			IAM_{high} (n=212) vs. IAM_{low} (n=1251)		
	**nGene**	**%**	**Gene-eQTL pairs AFR_{high}**	**nGene**	**%**	**Gene-eQTL pairs IAM_{high}**
Genes analyzed	9267	100	2,653,736	4587	100	783,676
No overlap in 95% credible set[1,2]	4405	45.8	1,116,628	1726	20.3	204,927
Tier 1	2620	27.3	39,300	280	3.3	2,263
Tier 2	111	1.2	111	5	0.1	5
Overlapping 95% credible set[3]	4862	50.6	1,537,018	2861	33.6	578,749
Tier 3	1	<0.001	1	0	0	0
**Union of Tiers 1-3**	**2701**	**28.1**	**39,412**	**284**	**3.3**	**2268**

[1] Tier 1 eQTLs includes variants that are rare (MAF<0.01) or monomorphic in the low ancestry (<10%) group

[2] Tier 2 eQTLs were identified using fine-mapping using PESCA and include variants with posterior probability (PP_{high})>0.80 of being specific to AFR_{high} or IAM_{high} and have PP_{high}>PP_{low}

[3] Tier 3 eQTLs show effect size heterogeneity based on Cochran's Q test (P_Q<0.05/number of genes tested)

Abbreviations

AFR	African ancestry
IAM	Indigenous American ancestry

Supplementary Table 13: Trans-eQTL discovery in GALA II/SAGE studies. Independent trans-eQTLs were identified using LD clumping within 1000 kb windows and $r^2<0.1$ for each gene. Trans-eQTL mapping and LD clumping was performed in groups stratified by self-reported race/ethnicity and genetic ancestry.

Population	Sample Size	Trans-eQTLs	Independent trans-eQTLs	eGenes
Pooled (Total)	2733	9864	647	414
African American	757	329	39	33
Puerto Rican	893	956	67	52
Mexican American	784	1168	62	51
AFR_{high}	721	283	36	31
Filtered[1]	721	149	26	24
IAM_{high}	610	691	26	22
Filtered[2]	610	350	23	20

[1] All trans-eQTLs detected in AFR_{low} group were removed

[2] All trans-eQTLs detected in IAM_{low} group were removed

Abbreviations

AFR	African ancestry
IAM	Indigenous American ancestry
Pooled	Analysis also includes participants self-identified as Other Latino

Supplementary Table 14: TWAS model performance. Cross-validation (CV) R^2 of gene expression prediction models generated by PredictDB. Heritability, CV R^2, and V_G are summarized across the final set of genes included in the TWAS models.

Population	Number of Genes			h^2	CV R^2	V_G
	Input[1]	Pass[2]	Final[3]			
Pooled (Total)	14,900	11,943	11,830	0.186	0.157	0.061
African American	15,012	10,782	10,090	0.246	0.180	0.077
Puerto Rican	14,756	10,039	9,611	0.212	0.163	0.071
Mexican American	14,893	9,665	9,084	0.205	0.167	0.062

[1] The total number of gene models generated from PredictDB

[2] Number of genes that passed the preliminary filters of CV correlation (rho_avg) > 0.1 and correlation z-score p-value < 0.05 for the correlation between predicted and measured gene expression values

[3] Number of genes with h^2 p-value < 0.05, the total number of genes with valid TWAS models

Abbreviations

AFR	African ancestry
IAM	Indigenous American ancestry
Pooled	Analysis also includes participants self-identified as Other Latino

Supplementary Table 15: Comparison of TWAS model performance with local ancestry (LA) adjustment. Cross-validation R^2 of gene expression prediction models with and without local ancestry adjustment. Comparisons were restricted to heritable genes with valid TWAS models.

Population	TWAS Models			CV R^2	
	**Original**	**LA-adjusted**	**Intersection**	**Original**	**LA-Adjusted**
Pooled (Total)	11830	11588	11497	0.161	0.154
African American	10090	9848	9701	0.186	0.177
Puerto Rican	9611	9090	8959	0.173	0.156
Mexican American	9084	8582	8475	0.177	0.161

Abbreviations

AFR	African ancestry
IAM	Indigenous American ancestry
Pooled	Analysis also includes participants self-identified as Other Latino
