# Supplementary material for: Gene expression in African Americans, Puerto Ricans and Mexican Americans reveals ancestry-specific patterns of genetic architecture
Source: Nat Genet. 2023 May 25;55(6):952–63. doi: 10.1038/s41588-023-01377-z (PMC10260401; doi:10.1038/s41588-023-01377-z)
Supplement: Supplementary file 2 — Reporting Summary [file 41588_2023_1377_MOESM2_ESM.pdf]

nature portfolio

Corresponding author(s): Elad Ziv

Last updated by author(s): 3/17/2023

Reporting Summary

Nature Portfolio wishes to improve the reproducibility of the work that we publish. This form provides structure for consistency and transparency in reporting. For further information on Nature Portfolio policies, see our Editorial Policies and the Editorial Policy Checklist.

Statistics

For all statistical analyses, confirm that the following items are present in the figure legend, table legend, main text, or Methods section.

n/a	Confirmed	
☐	☒	The exact sample size (*n*) for each experimental group/condition, given as a discrete number and unit of measurement
☐	☒	A statement on whether measurements were taken from distinct samples or whether the same sample was measured repeatedly
☐	☒	The statistical test(s) used AND whether they are one- or two-sided<br>*Only common tests should be described solely by name; describe more complex techniques in the Methods section.*
☐	☒	A description of all covariates tested
☐	☒	A description of any assumptions or corrections, such as tests of normality and adjustment for multiple comparisons
☐	☒	A full description of the statistical parameters including central tendency (e.g. means) or other basic estimates (e.g. regression coefficient) AND variation (e.g. standard deviation) or associated estimates of uncertainty (e.g. confidence intervals)
☐	☒	For null hypothesis testing, the test statistic (e.g. *F*, *t*, *r*) with confidence intervals, effect sizes, degrees of freedom and *P* value noted<br>*Give P values as exact values whenever suitable.*
☒	☐	For Bayesian analysis, information on the choice of priors and Markov chain Monte Carlo settings
☐	☒	For hierarchical and complex designs, identification of the appropriate level for tests and full reporting of outcomes
☐	☒	Estimates of effect sizes (e.g. Cohen's *d*, Pearson's *r*), indicating how they were calculated

Our web collection on statistics for biologists contains articles on many of the points above.

Software and code

Policy information about availability of computer code

Data collection	No software was used for data collection.
Data analysis	Open source codes used in this study:<br><br>GTEx FASTQTL docker image. Available at https://github.com/broadinstitute/gtex-pipeline/tree/master/qtl<br>GCTA Version 1.93.1beta. Available at https://cnsgenomics.com/software/gcta<br>CAVIAR v2.2. Available at https://github.com/fhormoz/caviar<br>PESCA version 0.3-beta. Available at https://github.com/huwenboshi/pesca<br>METASOFT v2.0.1. Available at http://genetics.cs.ucla.edu/meta<br>PredictDB v7 pipeline. Available at https://github.com/hakyimlab/PredictDB_Pipeline_GTEx_v7<br>MetaXcan version 0.7.4. Available at https://github.com/hakyimlab/MetaXcan<br>LDAK version 5.1 Available at https://dougspeed.com/ldak/<br>COLOC R package version 5.1 Available at https://cran.r-project.org/web/packages/coloc/index.html<br>Data visualization: ggplot2 (version 3.3.5), colorspace (version 2.0-2), ggrepel (version 0.9.1), ggfittext (version 0.9.1), cowplot (version 1.1.1); parts of Figure 1 and Supplementary Figure 1 were generated using BioRender (https://biorender.com)

For manuscripts utilizing custom algorithms or software that are central to the research but not yet described in published literature, software must be made available to editors and reviewers. We strongly encourage code deposition in a community repository (e.g. GitHub). See the Nature Portfolio guidelines for submitting code & software for further information.

Data

Policy information about availability of data

All manuscripts must include a data availability statement. This statement should provide the following information, where applicable:

- Accession codes, unique identifiers, or web links for publicly available datasets
- A description of any restrictions on data availability
- For clinical datasets or third party data, please ensure that the statement adheres to our policy

TOPMed WGS and RNA-seq data from GALA II and SAGE are available on dbGaP under accession number phs000920.v4.p2 and phs000921.v4.p1, respectively. TOPMed WGS data from SAPPHIRE are available under the dbGaP accession number phs001467.v1.p1. Individual-level normalized gene expression data for from GALA II and SAGE, models for performing transcriptome-wide association studies (TWAS), and eQTL summary statistics based on GALA II and SAGE are freely available from https://doi.org/10.5281/zenodo.7735723

Field-specific reporting

Please select the one below that is the best fit for your research. If you are not sure, read the appropriate sections before making your selection.

☒ Life sciences ☐ Behavioural & social sciences ☐ Ecological, evolutionary & environmental sciences

For a reference copy of the document with all sections, see nature.com/documents/nr-reporting-summary-flat.pdf

Life sciences study design

All studies must disclose on these points even when the disclosure is negative.

Sample size

2,733 individuals with available RNA samples with paired whole genome and RNA sequencing data were used.

Data exclusions

GALA II and SAGE recruitment: Participants were excluded if they reported any of the following: (1) 10 or more pack-years of smoking; (2) any smoking within 1 year of recruitment date; (3) history of lung diseases other than asthma (cases) or chronic illness (cases and controls); or (4) pregnancy in the third trimester.

Analyses in this manuscript were restricted to participants with whole genome sequencing (WGS) and RNA sequencing data generated by the NHLBI Trans-Omics for Precision Medicine (TOPMed) Program.

Samples were excluded if they failed sample quality control standards as described in the GTEx v8 documentation. Sample-level QC included removal of RNA samples with RIN < 6, genetically related samples (equal or more related than third degree relative), and sex-discordant samples based on reported sex and their XIST and RPS4Y1 gene expression profiles. Count distribution outliers were detected as follows: (i) Raw counts were normalized using the trimmed mean of M values (TMM) method in edgeR60 as described in GTEx v8 protocol. (ii) The log2 transformed normalized counts at the 25th percentile of every sample were identified (countq25). (iii) The 25th percentile (Q25) of countq25 was calculated. (iv) Samples were removed if their countq25 was lower than -4 as defined by visual inspection.

Replication

We assessed the out-of-sample performance of our gene expression prediction models using an independent African American study (SAPPHIRE) with RNA-seq data and confirmed our models generated prediction with higher correlation to measured gene expression levels than existing models.

Randomization

GALA II and SAGE are observational studies and did not have an intervention or randomization component. To account for hidden confounding factors in RNA-seq data such as batch effects, technical and biological variation in the sample preparation, and sequencing and/or data processing procedures, latent factors were estimated using the Probabilistic Estimation of Expression Residuals (PEER) method. Analyses adjusted for 50 to 60 PEER factors in analyses stratified by self-identified race/ethnicity and genetic ancestry. Inverse-normalized gene expression was regressed on PEER factors, and the residuals were used in downstream analyses. To ensure robust results, additional covariates included age at blood draw, sex, asthma case-control status, and the first 5 genetic ancestry principal components.

Blinding

Genomic DNA samples extracted from whole blood were sequenced as part of the Trans-Omics for Precision Medicine (TOPMed) whole genome sequencing (WGS) program and the Centers for Common Disease Genomes of the Genome Sequencing Program. WGS was performed at the New York Genome Center and Northwest Genomics Center WGS by researchers and technicians who were blinded to race/ethnicity and genetic ancestry of the participants, as well as their asthma case/control status. The researchers who carried out eQTL mapping and heritability analyses for this manuscript had no influence on how WGS and RNA-seq data were collected or processed.

Reporting for specific materials, systems and methods

We require information from authors about some types of materials, experimental systems and methods used in many studies. Here, indicate whether each material, system or method listed is relevant to your study. If you are not sure if a list item applies to your research, read the appropriate section before selecting a response.

Materials & experimental systems

n/a	Involved in the study
☒	☐ Antibodies
☒	☐ Eukaryotic cell lines
☒	☐ Palaeontology and archaeology
☒	☐ Animals and other organisms
☐	☒ Human research participants
☒	☐ Clinical data
☒	☐ Dual use research of concern

Methods

n/a	Involved in the study
☒	☐ ChIP-seq
☒	☐ Flow cytometry
☒	☐ MRI-based neuroimaging

Human research participants

Policy information about studies involving human research participants

Population characteristics

The Genes-environments and Admixture in Latino Americans II (GALA II) study and the Study of African Americans, Asthma, Genes & Environments (SAGE) include African American, Puerto Rican and Mexican American children between 8-21 years of age with or without physician-diagnosed asthma. Briefly, participants were eligible if they were 8-21 years of age and identified all four grandparents as Latino for GALA II or African American for SAGE.

Recruitment

Children were enrolled as a part of the ongoing Genes-environments & Admixture in Latino Americans (GALA II) case-control study. From July 2008 through November 2011, children were recruited from five centers (Chicago, Illinois; Bronx, New York; Houston, Texas; San Francisco Bay Area, California; and Puerto Rico) using a combination of community- and clinic-based recruitment. Participants were eligible if they were 8–21 years of age and all four grandparents self-identified as Latino. Asthma cases were defined as participants with a history of physician diagnosed asthma and the presence of two or more symptoms of coughing, wheezing, or shortness of breath in the 2 years preceding enrollment. Healthy controls were recruited from the community and clinics with the same catchment area as cases. Controls were defined as participants with no reported history of asthma, lung disease, or chronic illness over their lifetime, and no reported symptoms of coughing, wheezing or shortness of breath in the last two years. Controls were frequency matched on age (within 1 year), sex, and study center. Participants were excluded if they reported any of the following: (1) 10 or more pack-years of smoking; (2) any smoking within 1 year of recruitment date; (3) history of lung diseases other than asthma (cases) or chronic illness (cases and controls); or (4) pregnancy in the third trimester. All local institutional review boards approved the study, and all participants/parents provided appropriate written assent/consent.

The participant's country of birth, parents' country of birth, and self-reported country of birth of all four grandparents were used to determine country or region of origin. Firstly, for participants born outside the U.S., their origin was determined by their country of birth. For U.S.-born participants, origin was determined by their parents' country of birth (or grandparent's country of origin if their parents were born in the U.S.). For those with missing information for one parent, origin defaulted to the origin of the known parent. Participants were then classified as Puerto Rican, Mexican, South American, Central American, non-Puerto Rican Caribbean, or mixed Latino (for individuals of multiple origins who did not fit in the prior mentioned categories).

The Study of African Americans, Asthma, Genes, and Environments (SAGE II) is the largest ongoing gene-environment interaction study of asthma in African American children in the USA. SAGE II was initiated in 2006 and recruited participants with and without asthma until 2013 through a combination of clinic- and community-based recruitment centers in the San Francisco Bay Area. Institutional review boards of participant centers approved the study, and all participants or, for participants 17 or younger, their parents provided written informed consent. Participants 17 or younger also provided age-appropriate assent. Asthma cases were defined as individuals with a history of physician-diagnosed asthma and asthma controller or rescue medication use within the last 2 years and report of symptoms. Participants were eligible if they were 8–21 years of age and self-identified as African American and had four African American grandparents. Study exclusion criteria included the following: (1) any smoking within 1 year of the recruitment date, (2) 10 or more pack-years of smoking, (3) pregnancy in the third trimester, and (4) history of lung diseases other than asthma (cases) or chronic illness (cases and controls).

Ethics oversight

The local institutional review board from the University of California San Francisco Human Research Protection Program approved the studies (IRB# 10-02877 for SAGE and 10-00889 for GALA II). All subjects and their legal guardians provided written informed consent.

Note that full information on the approval of the study protocol must also be provided in the manuscript.
